# Monocyclic β-lactams loaded on hydroxyapatite: new biomaterials with enhanced antibacterial activity against resistant strains

**DOI:** 10.1038/s41598-017-02943-2

**Published:** 2017-06-02

**Authors:** Daria Giacomini, Paola Torricelli, Giovanna Angela Gentilomi, Elisa Boanini, Massimo Gazzano, Francesca Bonvicini, Emanuele Benetti, Roberto Soldati, Giulia Martelli, Katia Rubini, Adriana Bigi

**Affiliations:** 1 Department of Chemistry “G. Ciamician”, University of Bologna, Via Selmi 2, 40126 Bologna, Italy; 2 Laboratory of Preclinical and Surgical Studies, Codivilla-Putti Research Institute, Rizzoli Orthopaedic Institute, via di Barbiano 1/10, 40136 Bologna, Italy; 3 Dipartimento di Farmacia e Biotecnologie, University of Bologna Via Massarenti 9, 40138 Bologna, Italy; 4 Istituto per la Sintesi Organica e la Fotoreattività, ISOF-CNR, Via Gobetti 101, 40129 Bologna, Italy

## Abstract

The development of biomaterials able to act against a wide range of bacteria, including antibiotic resistant bacteria, is of great importance since bacterial colonization is one of the main causes of implant failure. In this work, we explored the possibility to functionalize hydroxyapatite (HA) nanocrystals with some monocyclic N-thio-substituted β-lactams. To this aim, a series of non-polar azetidinones have been synthesized and characterized. The amount of azetidinones loaded on HA could be properly controlled on changing the polarity of the loading solution and it can reach values up to 17 wt%. Data on cumulative release in aqueous solution show different trends which can be related to the lipophilicity of the molecules and can be modulated by suitable groups on the azetidinone. The examined β-lactams-HA composites display good antibacterial activity against reference Gram-positive and Gram-negative bacteria. However, the results of citotoxicity and antibacterial tests indicate that HA loaded with 4-acetoxy-1-(methylthio)-azetidin-2-one displays the best performance. In fact, this material strongly inhibited the bacterial growth of both methicillin resistant and methicillin susceptible clinical isolates of *S. aureus* from surgical bone biopsies, showing to be a very good candidate as a new functional biomaterial with enhanced antibacterial activity.

## Introduction

The increased life expectancy in developed countries has led to a serious rise in the number of age-related musculoskeletal disorders and hence, to an increasing demand of materials for the repair and substitution of damaged tissues, including orthopedic implants for joint replacement. At present, implant premature failures amount to about 10%, a number which will significantly increase in the next future due to the continuous aging of the population^1^. Aseptic loosening and infections represent the main causes of implant failure. Although aseptic surgical techniques and prophylactic systemic antibiotic therapy have significantly reduced infections, bacterial colonization of implants and medical devices is still a major problem. Microorganisms may colonize the implant through direct inoculation at the time of implantation or they may reach the implant by haematogenous seeding during bacteraemia or through direct contiguous spreading from an adjacent infectious focus^2, 3^. Early and delayed infections are usually acquired during implantation of the prosthesis, whereas late infections are predominantly acquired by haematogenous seeding^2^. After colonization, bacteria may adhere to the surface of the bone or to the orthopedic implants producing a self-protective biofilm, which exhibits remarkable resistance against adverse agents, such as the host immune systems and antibiotics^4^. This is also due to the intensive use of antibiotics, which has provoked bacterial resistance to many antimicrobial agents^5, 6^. Moreover, biofilms promote gene transfer between resistant and non-resistance microbial strains^7, 8^, and the systemic administration of very potent antibiotics can provoke irreversible damage to other organs^9^.

The problem, which often requires removal of the infected implant, has prompted a number of studies aimed to design and develop antimicrobial surface coatings and biomaterials, through functionalization with antibacterial agents and antibiotics. Most antimicrobial agents, such as silver, chlorhexidine and nitric oxide display adverse side effects and/or low efficiency, whereas the antimicrobial resistance of coatings containing classical antibiotics depends on the activity of the drug on resistant strains and on their side-effects^8, 10–12^.

**Figure 1:**
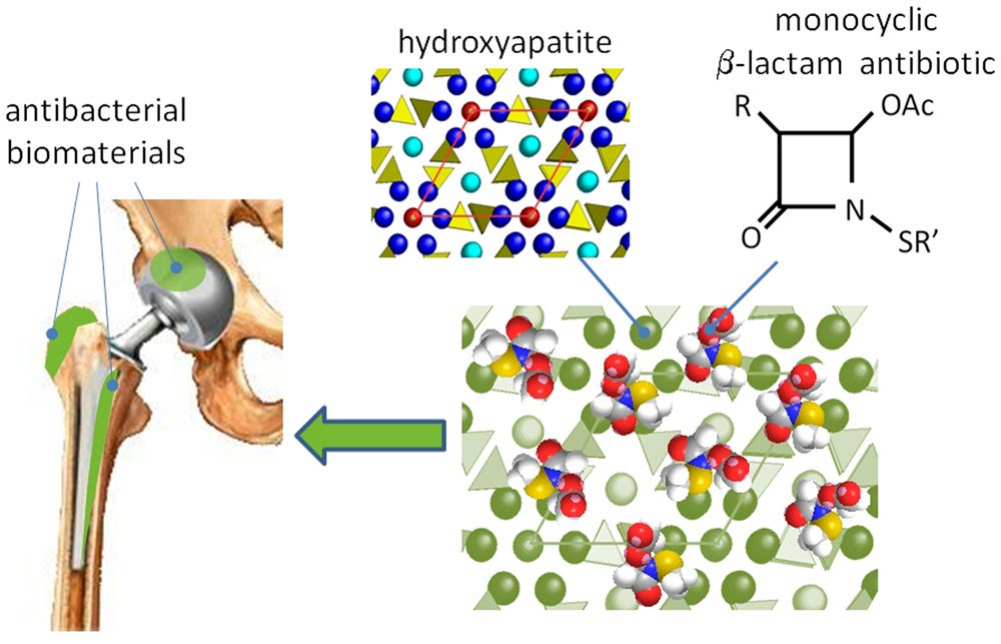
Sketch of the possible applications of the biomaterials developed in this paper. R and R’ are defined in Figure 3.

Due to their excellent biocompatibility and bioactivity, calcium orthophosphates are widely used for the preparation of biomaterials for hard tissues substitution and repair, including coatings for metallic implants, bone cements and scaffolds for regenerative medicine^13–15^. To this aim, the most employed calcium phosphate is hydroxyapatite (HA), thanks to its similarity to the inorganic phase of bone. The biological performance of HA can be improved through functionalization with biological relevant ions and molecules^16, 17^. In particular, HA functionalized with silver nanoparticles, or doped with silver, copper and zinc ions has been reported to display antibacterial activity towards Gram-positive and Gram-negative bacteria^18–20^. HA has been previously proposed also as support for classical antibiotics^21–23^ in order to obtain antibacterial materials without possible allergic reactions due to the presence of metal ions. In this paper, we have functionalized HA nanocrystals with a series of new monocyclic N-thio-substituted β-lactams with the aim to get new composite materials with relevant activity against a wide range of bacteria, including antibiotic resistant bacteria (Fig. 1).

β-lactam antibiotics are still the main class of agents used to treat bacterial infections^24^. Beside bicyclic β-lactam classes such as penicillins, cephalosporins and carbapenems, monocyclic compounds have emerged due to their interesting, variegated biological activities^25^. The efficacy evaluation against *Staphylococcus aureus*, including methicillin resistant strains (MRSA) of some monocyclic β-lactams with an alkylthio-group on the β-lactam nitrogen atom, has recently been reported^26–28^. Structure-activity relationship studies pointed out that the presence of a *N*-methylthio substituent proved to be essential for antimicrobial activity. The most active compounds showed minimum inhibitory concentration (MIC) values of 4 and 8 mg/L against MRSA isolated from pediatric patients with cystic fibrosis^27^.

The main goal of the present work is to study the loading of some monocyclic β-lactams on HA nanocrystals in order to get functionalized materials able to couple the bioactivity of HA with the antibacterial properties of the β-lactams. To this aim, we have carried out a chemical, structural, and morphological characterization of the new *N*-thio-azetidinone-functionalized hydroxyapatites, and evaluated them against Gram-positive and Gram-negative reference bacteria as well as antibiotic-resistant strains from clinical isolates obtained from surgical bone biopsies. In particular we selected isolates of *S. aureus*, including MRSA with small-colony variant (SCV) phenotype frequently associated to persistent infections, as they, together with *Staphylococcus epidermidis*, account for close to 65–80% of prosthetic joint infections^29, 30^. The new functionalized HA could be a good answer to the limited antibiotic options for an effective local control of MRSA bone infections which call for new anti-infective drugs to prevent and treat this human pathogen.

## Results and Discussion

### Synthesis of β-lactams

**Figure 2:**
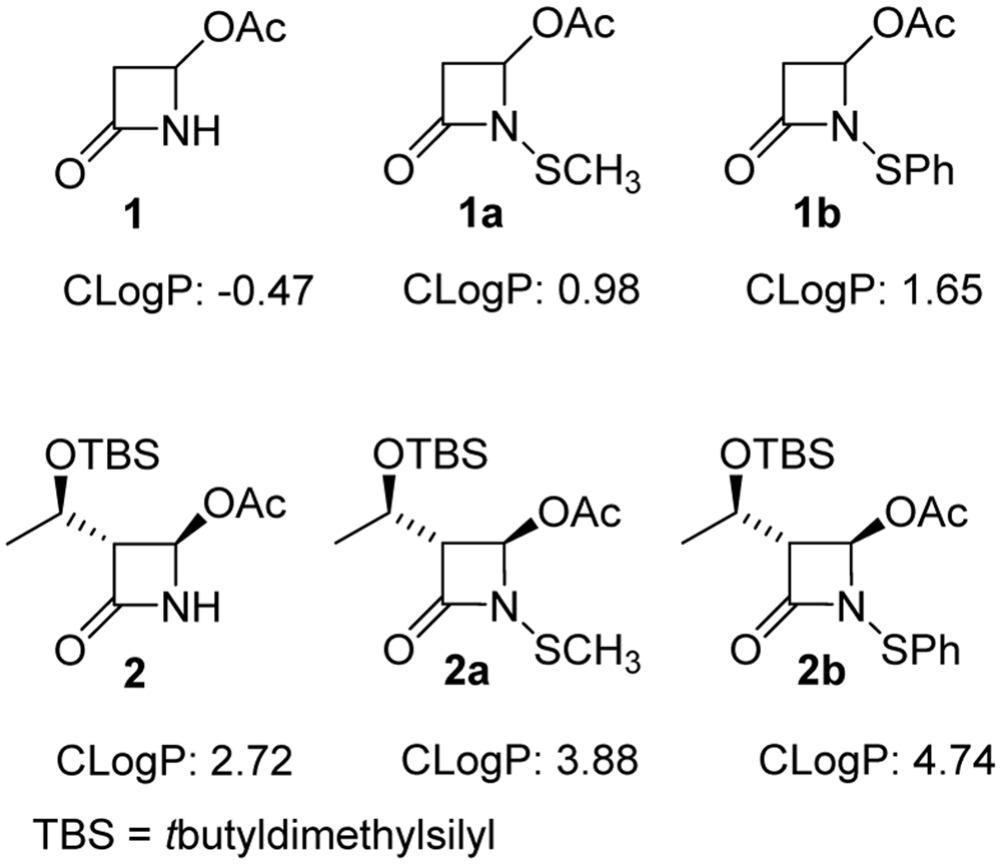
β-lactams evaluated in this study. Calculated logP (CLogP) values were obtained with ChemDraw 15.0 program (specific algorithms for calculating logP from fragment based methods were developed by the Medicinal Chemistry Project of CambridgeSoft and BioByte).

A series of six monocyclic β-lactams (azetidinones) was selected to study the loading on hydroxyapatite (HA), and they were classified accordingly to the core structures shown in Fig. 2. We chose azetidinones **1** and **2** as models for N-unsubstituted (NH) compounds, *N*-methylthio-azetidinones (*N-*SCH_3_) **1a** and **2a** because they previously showed an interesting antibacterial activity against resistant strains^27^, and *N*-phenylthio-azetidinones (NSPh) **1b** and **2b** as de novo compounds with an enhanced lipophilic character. The lipophilicity of a molecule can be expressed by the partition coefficient P between *n*-octanol and water^31^. The calculated parameter ClogP reported in Fig. 2 indicates that the selected azetidinones cover a rather wide range of lipophilicity: from the most hydrophilic molecule **1** (ClogP = −0.47) to the most lipophilic one, **2b** (ClogP = 4.74). It is important to highlight that the OTBS-hydroxyethyl side chain confers a stronger lipophilic character to **2**, **2a**, and **2b** with respect to **1**, **1a**, or **1b**.

**Figure 3:**
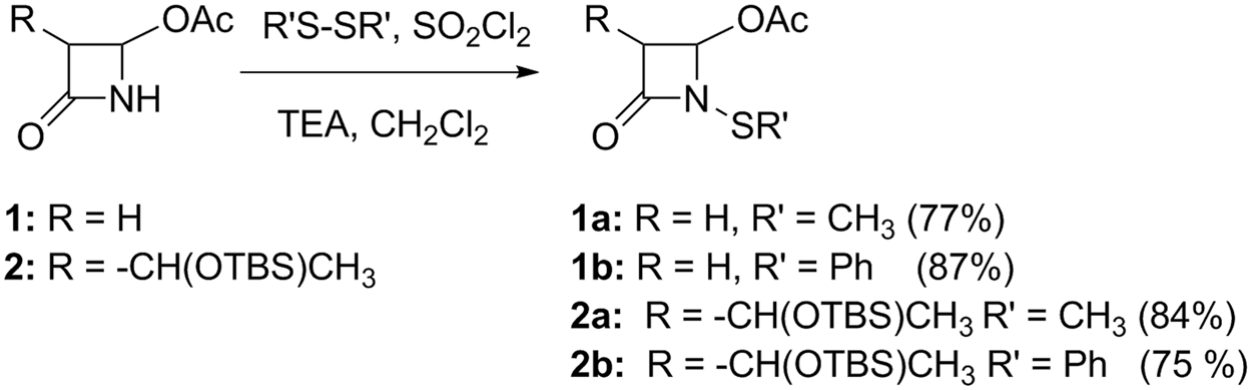
Synthesis of *N*-thiosubstituted β-lactams, reaction yields in parenthesis.

Azetidinones **1** and **2** are commercially available, **1a-b** and **2a-b** were synthesized according to the procedure depicted in Fig. 3.

At variance with that previously reported^27^, the new optimized *N*-methylthiolation procedure using dimethyl- or diphenyl-disulfide in the presence of sulfuryl chloride and triethylamine (TEA) in dichloromethane (Fig. 3) utilizes milder reaction conditions and the crudes are easily purified by flash chromatography. With this new procedure starting from compounds **1** and **2** using dimethyldisulfide or diphenyldisulfide, azetidinones **1a**, **2a**, **1b**, and **2b** were obtained in good yields, 77, 84, 87, and 75% isolated yields, respectively.

### Loading of azetidinones on HA

Nanocrystalline HA used as adsorption substrate in this study was synthesized as previously reported^32^ in a well crystallized single phase, as shown by its XRD pattern (Fig. SI-1), which displays only peaks belonging to calcium hydroxyapatite. The product is characterized by the cell parameters *a* = 9.428(2)Å, *c* = 6.881(1)Å, Ca/P = 1.66, Surface Area = 55 ± 5 m^2^/g. Synthetic HA is often employed in the shape of sintered coarse particles with crystal size and shape quite different from those of biological apatites, which are characterized by very small crystal dimensions. The HA synthesized in the present study is closer to bone mineral in crystal size and morphology. In fact, it is constituted by HA nanocrystals, which exhibit mean dimensions of about 200 × 40 nm^16^ and have been previously shown to promote osteoblast proliferation and differentiation^33^. Moreover, these nanocrystals have been demonstrated to stimulate endothelial cell functions and biochemical pathways, which suggests that they could be successfully employed to promote angiogenesis, and in turn to rouse appropriate osteogenesis^34^.

**Figure 4:**
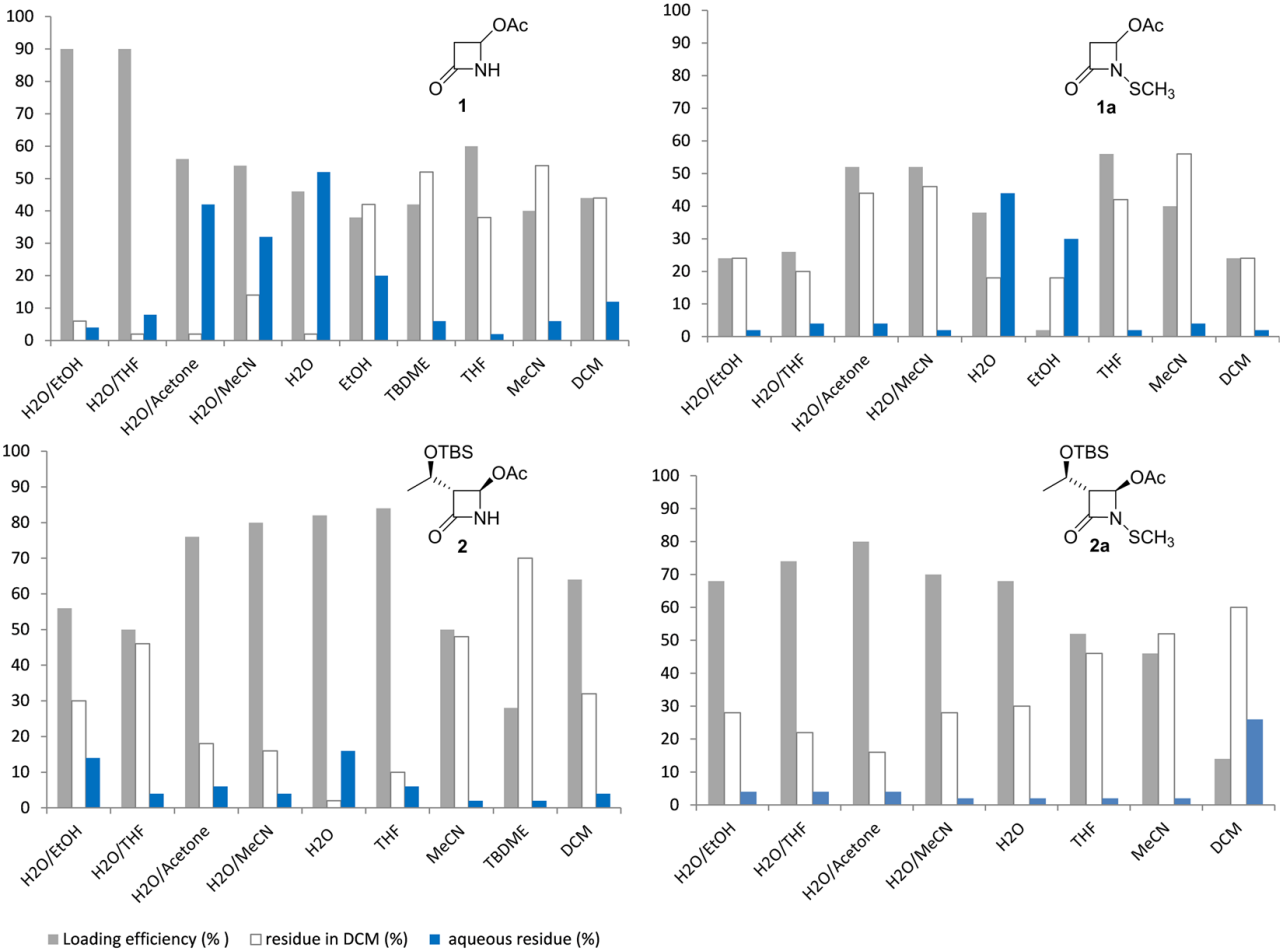
Medium effect on loading of azetidinones **1**, **1a**, **2**, and **2a** on HA. Loading efficiency % (grey), azetidinone residue in DCM (white) and in the aqueous layer (blue).

The adsorption study was performed at first on azetidinones **1**, **1a**, **2**, and **2a** chosen as models differentiated for polarity and solubility. The loading of azetidinones on HA was conducted in H_2_O or H_2_O/organic solvent 1:1 mixtures to study the effect of medium (see experimental section and SI). The solid functionalized HA samples were isolated and characterized. The supernatant aqueous solutions were extracted with dichloromethane (DCM). They were separately evaporated to quantify the amount of unloaded azetidinones in the two layers. Results were expressed as loading efficiency % (see formula in SI) and reported in Fig. 4.

The loading was found to be dependent on the azetidinones and on the medium. Compound **1** was efficiently loaded (90%) with H_2_O/ethanol or H_2_O/THF 1:1 mixtures, whereas **1a** was loaded at a maximum of 56% efficiency in THF or in mixtures H_2_O/acetone or H_2_O/CH_3_CN. Compound **2** was efficiently loaded in H_2_O, THF, or in aqueous mixtures as H_2_O/CH_3_CN or H_2_O/acetone; also **2a** was more efficiently loaded in water or aqueous mixtures. The mixture H_2_O/acetonitrile was chosen as standard solvent to load β-lactams **1a** and **2a** despite the good efficiency obtained also with H_2_O/acetone, in order to avoid the possible formation of autocondensation products of acetone promoted by HA^35^. Also the more lipophilic azetidinones **1b** and **2b** were loaded in H_2_O/CH_3_CN mixture with good efficiencies, 88% and 84%, respectively.

The dependence on concentration and on polarity of the loading solution was investigated on the antibacterial azetidinones **1a**, **1b**, and **2a**. Determination of the amount of the azetidinone loaded on HA was assessed by thermogravimetric analysis (TGA) on the dried **1a-HA**, **1b-HA**, and **2a-HA** samples and data were reported in Table 1. Examples of TGA scans are reported in Fig. SI-3.

**Table 1:** Effects of solution concentration and polarity on loading of azetidinones **1a**, **1b** and **2a** on HA. Loading was evaluated through TGA analysis.

Entry	Compound	Concentration (M)	Solvent		Loading	
			H_2_O/acetonitrile^a^	Polarity index^b^	wt %	mmol/g
1	**1a**	0.14	1:1	58.5	9.5 ± 0.7	0.54 ± 0.04
2	**1a**	0.11	1:1	58.5	5.9 ± 0.5	0.34 ± 0.03
3	**1a**	0.07	1:1	58.5	5.3 ± 0.5	0.30 ± 0.03
4	**1a**	0.06	1:1	58.5	5.3 ± 0.5	0.30 ± 0.03
5	**1a**	0.17	1.75:0.25	71.4	10.0 ± 0.7	0.57 ± 0.04
6	**1b**	0.15	1:1	58.5	12.7 ± 0.7	0.54 ± 0.03
7	**1b**	0.11	1:1	58.5	8.5 ± 0.6	0.36 ± 0.03
8	**1b**	0.06	1:1	58.5	8.9 ± 0.6	0.37 ± 0.03
9	**1b**	0.11	1.75:0.25	71.4	17.9 ± 0.8	0.75 ± 0.03
10	**2a**	0.15	1:1	58.5	5.5 ± 0.5	0.17 ± 0.02
11	**2a**	0.075	1:1	58.5	4.8 ± 0.5	0.14 ± 0.02
12	**2a**	0.060	1:1	58.5	5.8 ± 0.5	0.17 ± 0.02
13	**2a**	0.015	1:1	58.5	5.1 ± 0.5	0.15 ± 0.02
14	**2a**	0.075	1.5:0.5	69.3	11.0 ± 0.7	0.33 ± 0.02
15	**2a**	0.075	1.75:0.25	71.4	15.1 ± 0.8	0.45 ± 0.02
16	**2a**	0.075	0:1	37.0	5.0 ± 0.5	0.15 ± 0.02

^a^V/v ratio. ^b^The solution polarity was calculated from the Snyder polarity index of pure solvent^36^.

The range of concentrations was limited by the solubility of the respective azetidinones in the aqueous mixture. The loading of **1a** on HA was higher at 0.14 M (9.5 wt%), as indicated by TGA measurements of the corresponding **1a-HA** samples, but from 0.11 M to 0.06 M it remained almost constant (5.9–5.3 wt%, entries 1–4, Table 1). Azetidinone **1b** showed a similar behavior as **1a** with a 12.7 wt% at 0.15 M and 8.5–8.9 wt% at 0.11 and 0.06 M, respectively (entries 6–8, Table 1). On the contrary, the loading of **2a** was nearly independent on concentration (5.8–4.8 wt %, entries 10–13, Table 1).

Variation of the medium polarity was obtained on changing the composition of the H_2_O/acetonitrile mixtures in the loading solution. The solvent polarity index for each mixture was calculated from the Snyder polarity indexes (PI) of H_2_O and acetonitrile^36^. The loading was less affected by the medium polarity for the most hydrophilic azetidinone **1a** that ranges from 9.5 wt% in a 1:1 mixture of H_2_O/CH_3_CN to 10 wt% in a 7:1 ratio (entries 1 and 5, Table 1), whereas the loading was considerably affected by the polarity of the medium for the more hydrophobic compounds **1b** and **2a** (entries 6 and 9 for **1b**, entries 11 and 15 for **2a** Table 1). At constant concentration (0.075 M) of **2a** the loading on HA was triplicated from acetonitrile alone (PI = 37) to H_2_O/acetonitrile 7:1 mixture (PI = 71) (entries 15 and 16, Table 1). Thus the adsorption of the lipophilic azetidinone **2a** on HA was more favored from a water enriched loading solution.

This result could be explained in terms of effects on the solute solvation: an increase of water content in the loading solution destabilizes the solvation of hydrophobic **2a** in acetonitrile that, consequently, could be more efficiently taken up by the adsorbent HA^37, 38^. Thanks to this effect, the amount of azetidinone loaded on HA could be properly controlled by changing the relative amounts of the loading solution components.

In order to visualize the azetidinone adsorption onto HA, TBS group in compound **2** was substituted with a dansyl residue (**2Dan**) and then absorbed on HA with the above procedure to obtain the composite **2Dan-HA**. The observed fluorescence effect of **2Dan-HA** (Fig. SI-2) confirmed the presence of **2Dan** in the composite material.

## Characterization of HA samples loaded with azetidinones

All the composite samples showed similar XRD patterns as can be appreciated in Fig. SI-1; in particular, the patterns do not show any peak shifts compared to the starting HA suggesting that the crystal phase and the structure of the HA material is not affected by the presence of β-lactam molecules.

**Figure 5:**
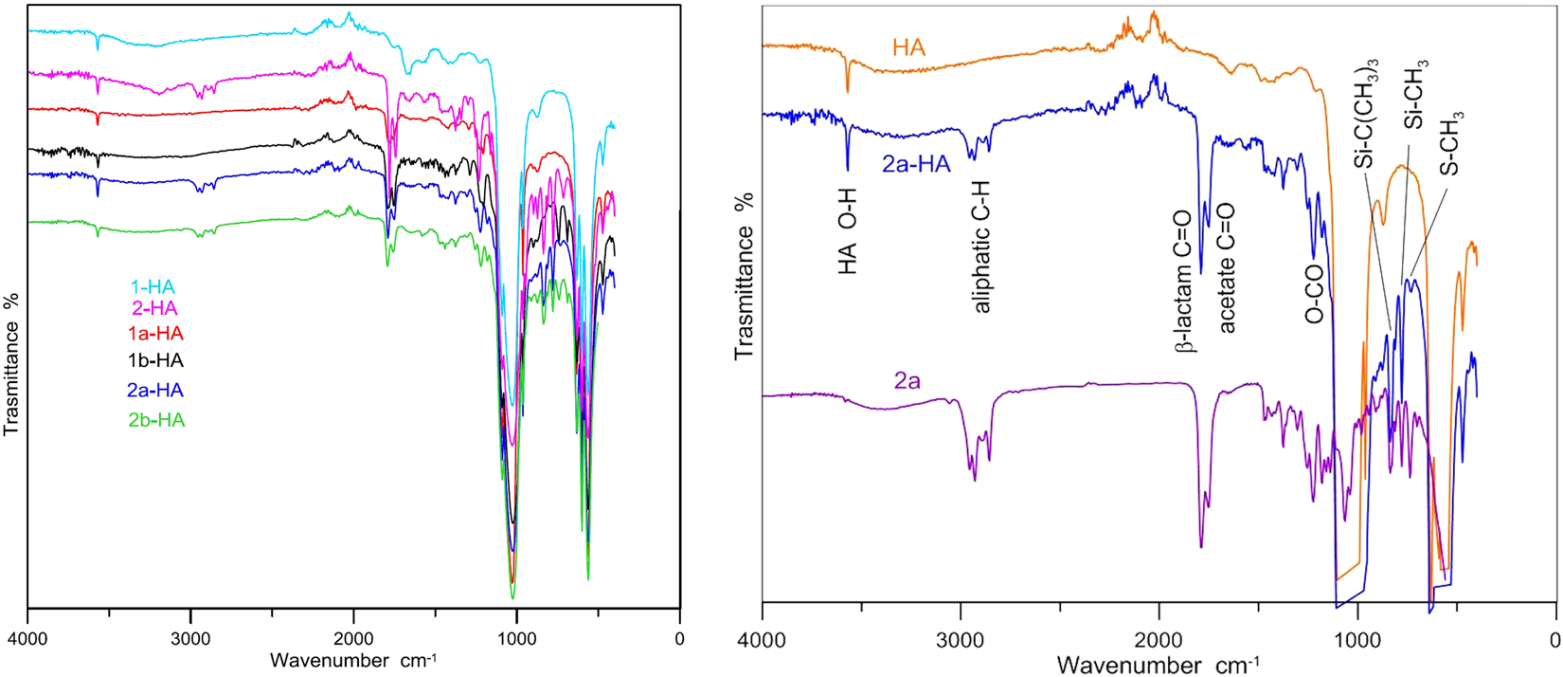
Left: ATR-FTIR spectra for samples **1-HA**, **2-HA**, **1a-HA**, **1b-HA**, **2a-HA**, **2b-HA**. Right: comparison between spectra of **2a-HA**, HA, and **2a** pure compound; assignments of the main bands are indicated. An enlarged view for all samples is reported in Fig. SI-4.

Figure 5 reports the ATR-FTIR spectra of the azetidinone-HA samples. The spectra display the O-H stretching and bending modes of hydroxyapatite at 3572 and 630 cm^−1^ respectively, the strong bands due to phosphate absorption in the 550–630 and 900–1100 cm^−1^ regions and the bands typical of the azetidinone molecules. In all the spectra the position of the azetidinone bands allows to recognize the specific functional groups. As an example, Fig. 5 (right) reports the band assignments for **2a-HA**: aliphatic C-H stretching vibrations in the 2900 cm^−1^ region, C=O stretching of β-lactam, acetoxy groups at 1790 and 1751 cm^−1^, respectively, and O-CO and Si-C stretching in the fingerprint region. Analysis of the FT-IR spectra thus revealed that the molecular integrity of the adsorbed azetidinones is fully preserved in the composites and no modification of the bands was observed upon absorption on HA.

It is worth to mention that intensities of azetidinones bands well correlate with the loading weight % determined by TGA analysis. As an example, the intensity of the C=O band at 1790 cm^−1^ shows a good linear correlation with the loading of a series of **2a-HA** samples (Fig. SI-5). Thanks to this correlation, ATR-FTIR analysis could be a fast method to evaluate the amount azetidinones loaded on HA.

The amount of β-lactam loaded on HA can reach relevant values with respect to other antibacterial agents loaded on apatites, providing a better covering of the material. On considering that the HA synthesized in this work has a surface area of 55 m^2^/g, it can be calculated, as an example, that **2a** is adsorbed up to 500 μmol/g (9 μmol/m^2^). This value is significantly higher than those reported for tetracycline loaded on biomimetic HA (82 μmol/g, 0.68 μmol/m^2^)^11^ and for ampicillin on HA (20 μmol/g)^39^.

Solid state ^1^H and ^13^C NMR spectroscopy analysis indicates that loading onto HA does not alter significantly the structure of β-lactams. As an example, resonance signals in ^1^H MAS NMR spectrum of **2a-HA** appeared at the same frequencies as those of **2a** in solution but with broader lines (see Fig. SI-6). Even the ^13^C MAS NMR of **2a-HA** showed the same signals as the molecule alone (Fig. SI-7), except for the C=O resonances. In the region 162–170 ppm, **2a-HA** presents two signals corresponding to the two C=O groups (169.2 and 168.9 ppm, ester and lactam), together with some broadened ones that could suggest the presence of different situations in which the C=O groups could be involved in non-covalent interactions with HA.

**Figure 6:**
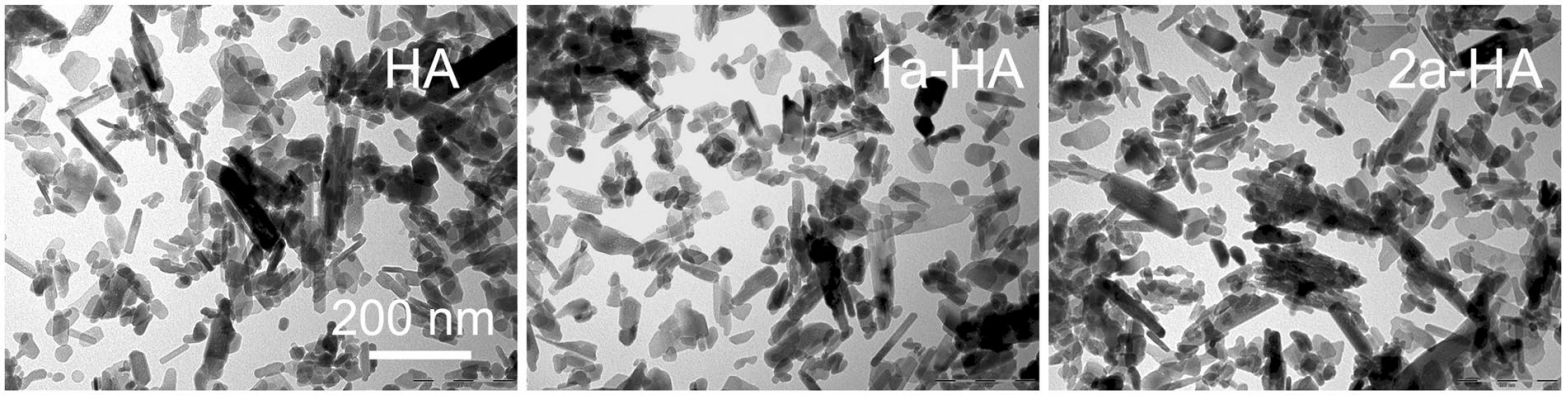
TEM images of HA, **1a-HA**, and **2a-HA** nanocrystals. Scale bar = 200 nm. All images have the same magnification.

TEM images of the different samples show that the azetidinone-HA composites are constituted of plate-like crystals, coherently with the ty’pical morphology of HA, which is characterized by crystals elongated along the *c*-axis direction. No significant morphological variation has been observed after azetidinone loading, as shown in Fig. 6 for samples **1a-HA** and **2a-HA** compared with pure HA.

### Antibacterial susceptibility testing against reference bacterial strains

The *in vitro* antibacterial activity was studied for **1a-HA**, **1b-HA**, **2a-HA**, and **2b-HA** samples (not for **1-HA** and **2-HA** because **1** and **2** are inactive as antibacterial agents)^27^. The antibacterial activity was examined by the KB disk diffusion test in which the area of clear media around the disk (Ø = 6 mm) indicates the degree of sensitivity of the strain expressed in millimeters. The reading and interpretation of the agar plates were made following 24 h of incubation at 37 °C. When zones of inhibition were present around the disks, plates were further observed for other 7 days, to notice any modifications in time, but the inhibitory effects remained constant. Table 2 reports the activities of compounds towards Gram-positive and a Gram-negative bacterial strains.

**Table 2:** Antibacterial activity: diameter of the inhibition zone (in mm) surrounding the azetidinone-HA samples against *S. aureus* and *E. coli* strains.

Sample	β-lactam content wt %	*S. aureus* ATCC 25923	*E. coli* ATCC 25922
1a-HA	8.1	30 ± 1	27 ± 1
1b-HA	17.0	27 ± 1	12 ± 1
2a-HA	14.0	20 ± 1	14 ± 1
2b-HA	16.5	16 ± 1	11 ± 1
HA^a^		NA^b^	NA^b^
GMN^c^		18 ± 1	19 ± 1

^a^Pure HA disks were used as negative controls. ^b^Bacterial-free zone not appearing.

^c^Disks containing gentamicin 10 µg (Oxoid SpA, Italy) were used as positive controls.

All experiments were performed on triplicate, on three different days.

All tested azetidinone-HA samples displayed a significant antibacterial activity against both the ATCC strains, particularly against *S. aureus*. Indeed the inhibition zone for all HA composites is bigger or equal to that of the positive control sample. All the samples showed activity also against *E. coli* even if they exhibited smaller active diameter values *vs* control, except for **1a-HA** that remarkably affected *E. coli* growth, yielding an inhibition zone wider than that obtained for gentamicin. The activities of **1a-HA** and **2a-HA** against *S. aureus* are in agreement with the activities exerted by the two molecules **1a** and **2a**, which showed MIC ranges of 32–64 mg/L *vs* MRSA, and of 32–64 and 8–32 mg/L *vs* MSSA, respectively^27^. Concerning the activity against Gram-negative bacteria, the free azetidinone **1a** had a MIC range of 32–64 mg/L, whereas **2a** was completely inactive^27^. The increased antibacterial activity of the composites **1a-HA** and **2a-HA** against *E. coli*, could be due to an inherent higher local concentration of the β-lactam on the solid HA, and consequently to a higher efficacy of the new functionalized HA materials.

### Azetidinone release studies

**Figure 7:**
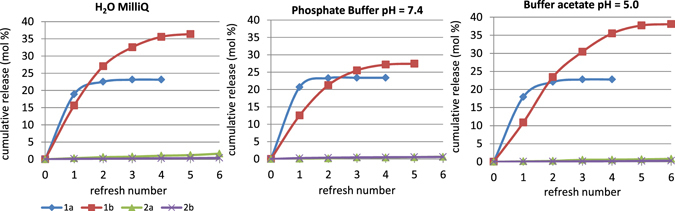
Release of azetidinones **1a** (♦ blue), **1b** (▪ red), **2a** (▴ green), and **2b** (**X** violet) from **1a-HA**, **1b-HA**, **2a-HA**, and **2b-HA**, in aqueous solution(left), buffer solution at pH 7.4 (center), buffer solution at pH 5.0 (right) media. The cumulative release is reported as mol %.

The *in vitro* release of azetidinones **1a**, **1b**, **2a**, and **2b** from the corresponding functionalized HA samples was evaluated by HPLC analysis. Three aqueous media were tested: deionized water (water MilliQ), phosphate buffer 0.1 M at pH = 7.4 as a model for a physiological pH condition, and acetate buffer 0.1 M at pH = 5 to mimic a pathological condition of a bacterial infection with a decreased pH due to the production of acidic metabolites by bacterial strains^40^. Results are reported in Fig. 7 and expressed as cumulative release in mol%. Early attempts showed scarcely detectable amounts of azetidinones released in the aqueous solutions which did not increase in the course of time (data not shown). It was then observed that only a refresh of the aqueous solution allowed a new release of the molecules. This fact could be due to the lipophilic character of these molecules which poorly desorbed from apatite because of their low affinity for the aqueous solution. Thus, the release data were reported as cumulative amounts over the refresh number. The release profiles of **1a** and **1b** in the three aqueous media showed a sort of initial burst release followed by a slower steady profile. The **1a-HA** sample released about 22% of the initial content of **1a** in the first two refreshes, with a low influence of the pH. Considering the steady profile, it could be estimated that **1a-HA** released 10^**−7**^ mol (around 17 μg) of **1a** per refresh. Interestingly, the release of **1b** was higher in acidic conditions thus affording a favorable pH responsiveness in case of bacterial infections. The initial burst of **1a-HA** and **1b-HA** could be related to that portion of molecules adsorbed on the surface in direct contact with the aqueous medium, whereas those molecules that interact more strongly with HA are progressively released during the steady state.

The release of azetidinones **2a** and **2b** is slower, probably due to their lower hydrophilicity (see ClogP values in Fig. 2) that provides a slow diffusion in the aqueous solution. From the collected data, it could be estimated a release in H_2_O MilliQ of about 3 μg per refresh for **2a**, and 4 ng per refresh for **2b**. It is important to underline that a low release could be favorable for the maintenance of an active concentration of the molecule on HA thus supplying an efficient antibacterial activity for a longer period.

The release of azetidinones **1a** and **2a** in buffer acetate (pH = 5) was further on followed with a once-a-day refresh for 9 days. After two days the **1a-HA** sample released about 22 mol% of the whole **1a** loaded on HA (initial burst release), in the next 6 days only traces per day were released. The **2a-HA** sample showed a sustained release of 0.4 mol% per day.

### Cytotoxicity tests

The cytotoxicity of samples **1a-HA**, **1b-HA**, **2a-HA**, **2b-HA** and unloaded **HA** as reference was tested using MG63 osteoblast-like cell line, widely employed for biomaterial testing with good responsiveness in *in vitro* studies of cell-material interaction. In the present study MG63 were cultured in direct contact with the samples.

**Figure 8:**
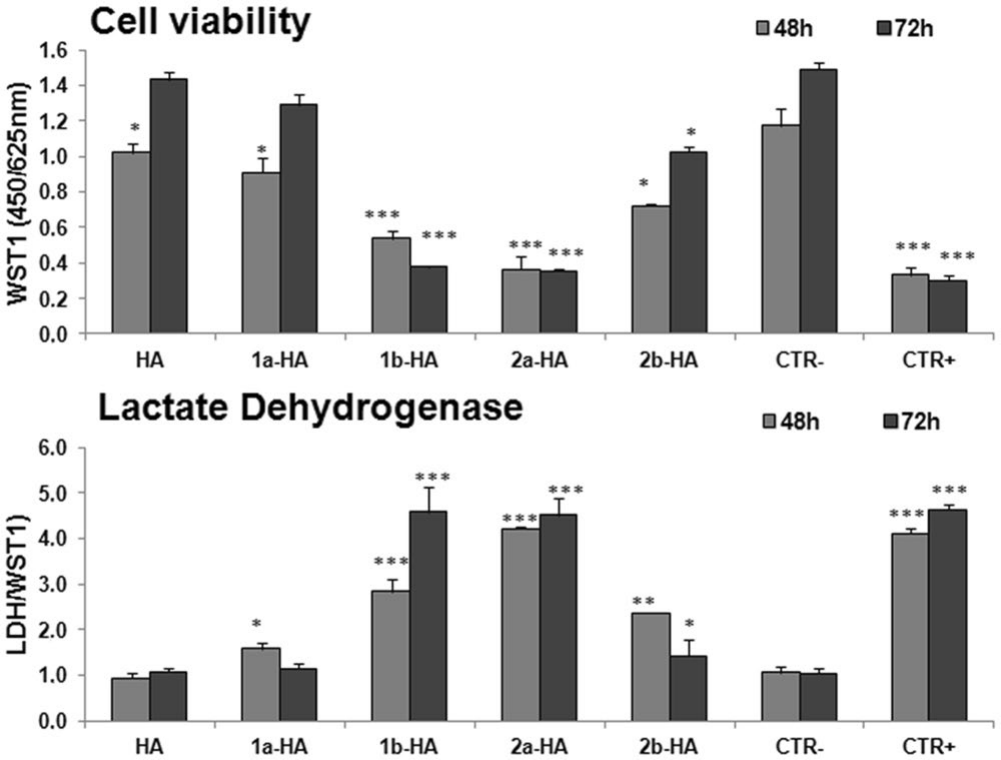
WST1 assay (a), and LDH release (b) of MG63 after 48 and 72 hours of culture on **HA**, **1a-HA**, **1b-HA**, **2a-HA**, **2b-HA** samples and CTRs. Values are reported as mean ± SD (*p < 0.05; **p < 0.005; ***p < 0.0005). (a) ***CTR + , 1b-HA, 2a-HA *vs* **HA**, **1a-HA**, **2b-HA**, **CTR–** (48 and 72 h); *1a-HA *vs* **HA**, CTR- (48 h); *2b-HA *vs* **HA**, CTR- (48 and 72 h), *HA *vs* CRT– (48 h**)**. (b) ***CTR + , **1b-HA**, **2a-HA** *vs* **HA**, **1a-HA**, **CTR–** (48 and 72 h); *1a-HA *vs* **HA**, CTR- (48 h); **2b-HA *vs* **HA**, CTR- (48 h); *2b-HA *vs* CTR- (72 h).

WST1 assay results at 48 and 72 h of culture are reported in Fig. 8. Values under 70% indicate cytotoxicity of tested material (UNI EN ISO 10993-5). HA as reference material showed lower viability in comparison with CTR– at 48 h, but no differences where found at 72 h; therefore HA demonstrated no signs of cytotoxicity, as percentage of viability at 48 and 72 h was 87% and 96% respectively. **1b-HA**, **2a-HA**, and **2b-HA** samples showed significant lower proliferation when compared to CTR– and **HA** at both 48 and 72 h, and their percentage of viability was always under 70%, index of cytotoxic effects (48 h: 46%, 31%, 61%; 72 h: 25%, 24%, 69% respectively). On the contrary cells grown in contact with **1a-HA** showed significant lower values at 48 h, but at 72 hours no significant difference in comparison with CTR- and **HA** was found. Percentage of viability of **1a-HA** was over 70% both at 48 and 72 h (77% and 86% respectively).

LDH is released in culture medium by cells with damaged membranes. The values measured in supernatants showed significant higher release in **1b-HA, 2a-HA** groups in comparison to **HA** and CTR- at both experimental times. Values for **1a-HA** and **2b-HA** were higher at 48 h, but they does not differ from **HA** (both **1a-HA** and **2b-HA**) or CTR- (**1a-HA**) at 72 h. Statistical analysis demonstrated that results of LDH were consistent and inversely correlated to WST1 proliferation assay (Pearson correlation coefficient −0.931, p < 0.005).

### Antibacterial activity against clinical isolates

From cytotoxicity evaluations of the new functional HA materials, **1a-HA** emerged as that having the best profile, so that its antibacterial efficacy was further assayed towards 10 clinical isolates (5 MSSA and 5 MRSA-SCV strains) all obtained from surgical bone biopsies, and representative of bacterial strains currently encountered during osteomyelitis. The 5 MSSA strains were susceptible to clindamycin, erythromycin, levofloxacin, oxacillin, penicillin, tetracycline and trimethoprim/sulfamethoxazole following the EUCAST susceptibility testing guidelines^41^. The 5 MRSA with SCV phenotype were resistant to oxacillin and other β-lactams; they were included in the present study as these strains are the main responsible for chronic and therapy-refractory infections despite systemic antimicrobial treatments due to their reduced rate of metabolism, intracellular persistence, strong adhesion to implants and host tissues via biofilm extracellular matrix formation^42^. Table 3 reports the diameters (in mm) of clearance zones surrounding the **1a-HA** samples. Data indicate that **1a-HA** strongly inhibited the bacterial growth of all the selected clinical isolates independently of methicillin resistant or susceptible *S. aureus*.

**Table 3:** Antibacterial activities against clinical isolates.

Clinical isolate	1a-HA	GMN*
**MSSA 1**	38 ± 1	30 ± 1
**MSSA 2**	28 ± 1	22 ± 1
**MSSA 3**	28 ± 1	20 ± 1
**MSSA 4**	33 ± 1	22 ± 1
**MSSA 5**	30 ± 1	20 ± 1
**MRSA-SCV 1**	30 ± 1	20 ± 1
**MRSA-SCV 2**	28 ± 1	21 ± 1
**MRSA-SCV 3**	34 ± 1	24 ± 1
**MRSA-SCV 4**	25 ± 1	21 ± 1
**MRSA-SCV 5**	30 ± 1	20 ± 1

^*^Disks containing gentamicin 10 µg (Oxoid SpA, Italy) were used as positive controls. All experiments were performed on duplicate in different days.

A similar activity on both MRSA and MSSA strains was previously observed on the free azetidinone **1a** ^27^. The corresponding result now obtained for the new **1a-HA** material confirms some hypotheses on the mechanism of action of these N-thiolated-azetidinones, in particular it excludes the possibility that the Penicillin Binding Protein PBP2a, which is the resistance factor discriminant between MRSA and MSSA, could be the biological target. Turos and colleagues investigated and discussed a tentative mechanism of action for N-thiolated-azetidinones^43^. Their hypothesis is that these derivatives could primarily block type II fatty acid biosynthesis in *S. aureus* through an initial transfer of the N-alkylthio moiety from the azetidinone nitrogen atom onto coenzyme A (CoA) to produce an alkyl-CoA mixed disulfide species, which then interferes with fatty acid biosynthesis, a novel essential target for the discovery of new antimicrobial agents. It is important to highlight that the loading of the N-thiolated-azetidinones on HA, as for **1a-HA**, does not interfere at all in the mechanism at the root of its antibacterial action.

## Conclusions

In this study we realized the loading of monocyclic β-lactams with a lipophilic character on nanocrystalline hydroxyapatite. The azetidinones showed high affinity interactions with HA and could be loaded in good amounts by a proper choice of the polarity of the loading solution. The characterization of the HA composites revealed the presence of intact β-lactams which could exert their biological activities. The release of azetidinones from HA composites depends on the lipophilicity of the molecule. The more lipophilic ones showed a low release which allows to maintain high local concentration of the antibacterial agent along the metabolic cycle of HA. Functionalization of biomimetic apatites by antibacterial agents active against resistant bacterial strains is important for the elaboration of bioactive bone-repair materials. All the new azetidinone-HA composites were successfully tested for antibacterial activity against reference strains. In particular, **1a-HA** sample, which displayed no cytotoxicity towards MG63 osteoblast-like cell line, showed excellent potency against a set of MSSA and MRSA-SCV clinical isolates of *S. aureus* obtained from surgical bone biopsies. Indeed combination of methicillin resistance and SCV phenotypic trait is a real threat for infected patients because they are difficult to remove from host tissues. The good results here obtained lead to new functional apatites with enhanced antibacterial properties able to prevent bone infections by resistant pathogens.

## Materials and Methods

An extended Materials and Methods chapter containing more experimental details and full spectroscopic data is reported in the Supplementary Information file.

### General Methods

All chemicals and solvents were of analytical grade; anhydrous solvents were obtained commercially and used without further drying. Materials were characterized by means of ATR-FTIR, TLC, HPLC-MS, ^1^H and ^13^C NMR, TGA, XRD, TEM investigations.

### Synthesis of hydroxyapatite

It was carried out using CO_2_-free distilled water in N_2_ atmosphere by dropwise addition of a (NH_4_)_2_HPO_4_ solution into a Ca(NO_3_)_2_ 4H_2_O solution^32^.

### Synthesis of azetidinones

Azetidinones **1** and **2** are commercially available (Sigma-Aldrich) and used as such.

#### 4-Acetoxy-1-(methylthio)-azetidin-2-one (**1a**)

In a 50 mL 2-neck flask under nitrogen, Me_2_S_2_ (113 μL, 1.25 mmol) was added to anhydrous dichloromethane (DCM, 1 mL). The mixture was stirred at 0 °C and a solution of SO_2_Cl_2_ (122 μL, 1.5 mmol) in anhydrous DCM (1 mL) was then added. After 15 min 4-acetoxy-azetin-2-one (129 mg, 1 mmol) was introduced followed by the addition of trimethylamine (TEA, 279 μL, 2 mmol). The mixture was stirred at reflux for 2 h. The consumption of the starting material was monitored by TLC analysis. After quenching, extraction, drying, concentration and LC purification, the pure product was obtained as a yellow oil in a 77% yield.

#### 4-acetoxy-1-(phenylthio)-azetidin-2-one (**1b**)

In a 25 mL 2-neck flask under nitrogen, Ph_2_S_2_ (218 mg, 1 mmol) in anhydrous DCM (1 mL) was introduced. The mixture was stirred at 0 °C and a solution of SO_2_Cl_2_ (122 μL, 1.5 mmol) in anhydrous DCM (1 mL) was then added. After 15 min 4-acetoxy-azetidin-2-one (129 mg, 1 mmol) was introduced followed by the addition of TEA (279 μL, 2 mmol). The mixture was stirred at reflux for 2 h. When TLC analysis indicated complete consumption of the starting material, the reaction was stopped and worked up to finally afford the product in a 87% yield (yellow oil) after LC purification.

#### (2*R*, 3*R*)-3-(-1-(*t*-butyldimethysilyloxy)ethyl)-4-acetoxy-1-(methylthio)-azetidin-2-one (**2a**)

In a 50 mL 2-neck flask under nitrogen, Me_2_S_2_ (90 μL, 1 mmol) was introduced in anhydrous DCM (1 mL). The mixture was stirred at 0 °C and a solution of SO_2_Cl_2_ (41 μL, 0.5 mmol) in anhydrous DCM (1 mL) was then added. After 15 min **2** (287 mg, 1 mmol) was introduced followed by TEA addition (307 μL, 2.2 mmol). The mixture was stirred at reflux for 2 h. After the consumption of the starting material, work-up and LC purification afforded **2a** as yellow oil in a 84% yield.

#### (2*R*,3*R*)-3-(-1-(*t*-butyldimethysilyloxy)ethyl)-4-acetoxy-1-(phenylthio)-azetidin-2-one (**2b**)

In a 25 mL 2-neck flask under nitrogen Ph_2_S_2_ (218 mg, 1 mmol) in anhydrous DCM (1 mL) was introduced. The mixture was stirred at 0 °C and a solution of SO_2_Cl_2_ (122 μL, 1.5 mmol) in anhydrous DCM (1 mL) was then added. After 15 min compound **2** (287 mg, 1 mmol) was introduced followed by the addition of TEA (279 μL, 2 mmol). The mixture was stirred at reflux for 2 h. When TLC analysis revealed the consumption of the starting reagent, the reaction was worked up to obtain **2b** product (dark yellow oil) with a 75% yield after LC purification.

### Azetidinone loading

The loading of azetidinones on HA was conducted in H_2_O (method A) or H_2_O/organic solvent mixtures (method B). Loading processes were set up in a parallel-synthesis fashion with a Carousel 6 reaction station using two necks round bottom flasks (50 mL). Reaction mixtures were controlled via TLC on the supernatant solution to monitoring the starting azetidinone disappearance. *Method A:* 200 mg of HA nanoparticles were suspended in 2 mL of H_2_O and warmed at 40 °C under magnetic stirring. Azetidinone (50 mg) was added in one portion to the suspension which was then heated up to 70 °C. After 4 h the mixture was quantitatively transferred with 1 mL of H_2_O/MeCN (1:1) in an open test tube and centrifugated for 1 min at 700 rpm and the solid phase was completely separated by the supernatant. The supernatant aqueous phase was collected and extracted with dichloromethane (1 × 3 mL). The aqueous and dichloromethane phases were separately evaporated and analyzed to quantify the unloaded azetidinone and its distribution in the two phases. The solid functionalized HA material was oven dried at 35 °C for 24 h, and kept in dessicator (CaCl_2_) for 24 h before the analyses. *Method B:* 200 mg of HA nanoparticles were suspended in 1 mL of H_2_O and warmed at 40 °C under magnetic stirring, then azetidinone (50 mg) was solubilized in 1 mL of organic solvent and added to the suspended HA, then the mixture was heated at 70 °C under stirring for 4 h. The work-up procedure was the same as for Method A.

Loading amount of the azetidinone molecules on HA was evaluated through thermogravimetric analysis as difference between the total weight loss measured between 38 and 800 °C for each loaded sample and that measured for pristine HA. Moreover, the determination was also performed through the evaluation of the intensity of the adsorption band of C=O at 1790 cm^−1^

### *In vitro* release

The release profiles of azetidinones loaded on HA were investigated in H_2_O Milli-Q, buffer phosphate (0.1 M, pH = 7.4), and buffer acetate (0.1 M, pH = 5). Samples of azetidinones **1a-HA** (8.1% of loaded azetidinone, TGA measurement), **1b-HA** (12.6%), **2a-HA** (15.7%), and **2b-HA** (10.85%) were used for the release study. In a 10 mL test tube an azetidinone-HA sample (50 mg) was suspended in 2.5 mL of the aqueous solutions (H_2_O Milli-Q, buffer phosphate, or buffer acetate). Experiments were conducted at 37 °C in thermostat with sampling and refresh of the aqueous solution after 1, 2, 3, 6, 8, 24, 30 h. At each time point, the solution was centrifugated (1 min. 700 rpm) the supernatant was separated and the released concentration of the azetidinone was determined by HPLC-UV analysis. The solid was incubated again with a fresh solution of the specific medium (2.5 mL). Linear calibration curves for the HPLC-UV analysis of azetidinones in supernatant solutions were established at 210 nm (column parameters and R_t_ in SI section). The release of samples **1a-HA** and **2a-HA** were also studied in buffer acetate at pH = 5 for 9 days by a once-a-day refresh with the procedure and analysis as above described.

### *In vitro* cytotoxicity

MG63 osteoblast-like cells were plated at a density of 2 × 10^4^ cells/mL in 24-well plates onto sterile samples of HA loaded with the monocyclic azetidinones **1a-HA**, **1b-HA**, **2a-HA**, **2b-HA**, unloaded HA as reference and in wells for negative (CTR−, DMEM only) and positive (CTR + , DMEM + 0.05% phenol solution) controls for cytotoxicity tests (according to UNI EN ISO 10993-5). After 48 and 72 h of culture cell proliferation and viability was assessed by WST1 (WST1, Roche Diagnostics) colorimetric reagent test. and the supernatant was collected to detect Lactate Dehydrogenase (LDH, enzyme-kinetic test, Roche Diagnostics) release: The statistical evaluation of data was performed using the software package SPSS/PC + StatisticsTM 23.0 (SPSS Inc., Chicago, IL).

### Antibacterial susceptibility testing

*Bacterial strains*. The *in vitro* effect of the HA nanocrystals loaded with the monocyclic azetidinones was evaluated against Gram-positive and Gram-negative reference bacterial strains: *Staphylococcus aureus* (ATCC 25923), *Escherichia coli* (ATCC 25922). In addition, clinical isolates obtained from surgical bone biopsies were included in the study and they were categorized based on their antimicrobial susceptibility to methicillin. The tested strains were isolated on BD Columbia Agar with 5% sheep blood (Becton Dickinson, Germany) and confirmed by MALDI-TOF MS (Bruker Daltonik, Germany)^44^. Their susceptibility was analyzed by the Vitek2 semi-automated system (bioMerieux, France) and interpreted following EUCAST guidelines. MRSA strains were confirmed by growth on BD oxacillin screen agar (Becton Dickinson, Germany), as in the clinical microbiology laboratory resistance to oxacillin is the marker for detecting methicillin resistance^45^. SCV phenotypic characterization was carried out by identification of very small pinpoint colonies on blood agar plate following 48 h of growth.

#### Kirby-Bauer (KB) disk diffusion method

The assay was performed following the requirements of the CLSI 2006) and allowed to measure the diameter of the inhibition zone (in millimeter) surrounding the azetidinone-HA samples. Briefly, the surface of MH agar (MHA) (Sigma-Aldrich) was inoculated with a bacterial suspension at 0.5 McFarland, prepared in sterile 0.9% saline solution. Gamma rays sterilized disk samples were placed on the agar plates and incubated at 37 °C for 24 hours when the reading and interpretation of zones of inhibition were carried out. Plates were further incubated up to 7 days to check the inhibitory effects over the time.

## Electronic supplementary material

Supplementary Information
