# Supplementary material for: Monocyclic β-lactams loaded on hydroxyapatite: new biomaterials with enhanced antibacterial activity against resistant strains
Source: Sci Rep. 2017 Jun 2;7:2712. doi: 10.1038/s41598-017-02943-2 (PMC5457414; doi:10.1038/s41598-017-02943-2)
Supplement: Supplementary file 1 — Supplementary Information [file 41598_2017_2943_MOESM1_ESM.pdf]

Supplementary Information

Monocyclic β-lactams loaded on hydroxyapatite: new biomaterials with enhanced antibacterial activity against resistant strains.

Authors:
Daria Giacomini[a], Paola Torricelli[b], Giovanna Angela Gentilomi[c], Elisa Boanini[a], Massimo Gazzano[d], Francesca Bonvicini[c], Emanuele Benetti[a], Roberto Soldati[a], Giulia Martelli[a], Katia Rubini[a], Adriana Bigi[a]

Affiliations:
[a] Department of Chemistry "G. Ciamician", University of Bologna, Via Selmi 2, 40126 Bologna, Italy.
[b] Laboratory of Preclinical and Surgical Studies, Codivilla-Putti Research Institute, Rizzoli Orthopaedic Institute, via di Barbiano 1/10, 40136 Bologna, Italy
[c] Department of Pharm. & Biotechnol., University of Bologna Via Massarenti 9, 40138 Bologna, Italy
[d] Istituto per la Sintesi Organica e la Fotoreattività, ISOF-CNR, Via Gobetti 101, 40129 Bologna, Italy

SUMMARY

1. Figures

Figure SI-1 XRD scans pag.........2
Figure SI-2 UV irradiation pag.........2
Figure SI-3 TGA scans pag.........3
Figure SI-4A ATR-FTIR expanded scans pag.........4
Figure SI-4B ATR-FTIR expanded scans pag.........5
Figure SI-5 Plot weight% *vs* IR absorption pag.........6
Figure SI-6 ^{1}H MAS-NMR spectra pag.........7
Figure SI-7 ^{13}C MAS-NMR spectra pag.........8

2. Material and Methods (extended version)

2.1 General Methods pag.........9
2.2 Synthesis of hydroxyapatite pag.........9
2.3 Synthesis of azetidinones (with spectroscopic data) pag.........9
2.4 Azetidinone loading pag.........11
2.5 In vitro release pag.........12
2.6 Cytotoxicity pag.........13
2.7 Antibacterial susceptibility testing pag.........13
2.8 References pag.........14

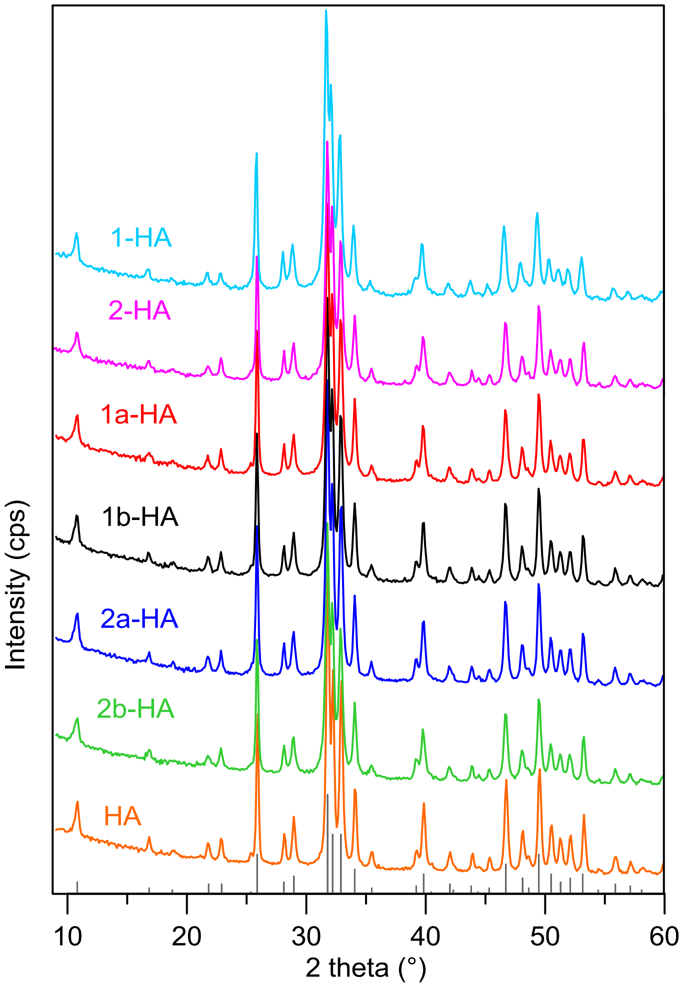

Figure SI-1. X-ray diffraction patterns of HA and composite samples: **1-HA**, **2-HA** , **1a-HA**, **1b-HA**, **2a-HA**, **2b-HA**. The vertical bars at the bottom display the reference pattern of calcium hydroxyapatite (International Centre for Diffraction Data file n. 9-432).

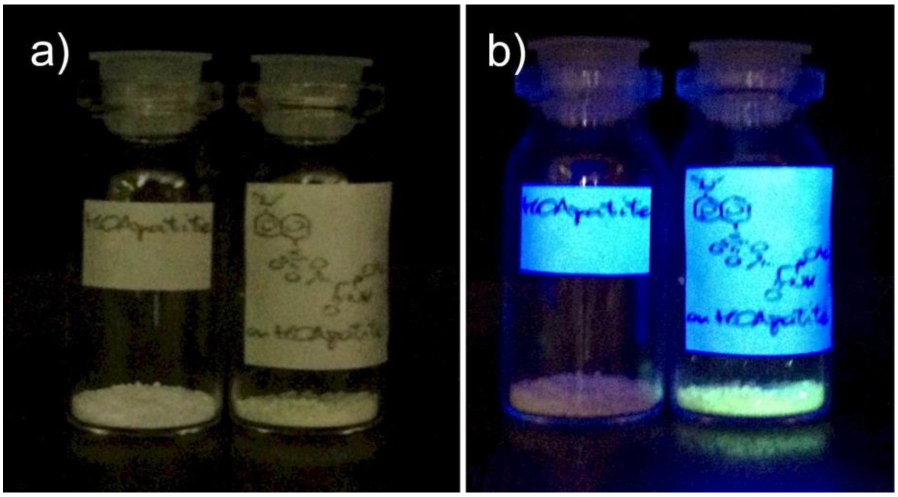

Figure SI-2. Effect of the UV irradiation (365 nm): light off (a), light on (b). In each picture: HA (left sample) and HA loaded with a β-lactam molecule containing dansyl derivative **2Dan-HA** (right sample).

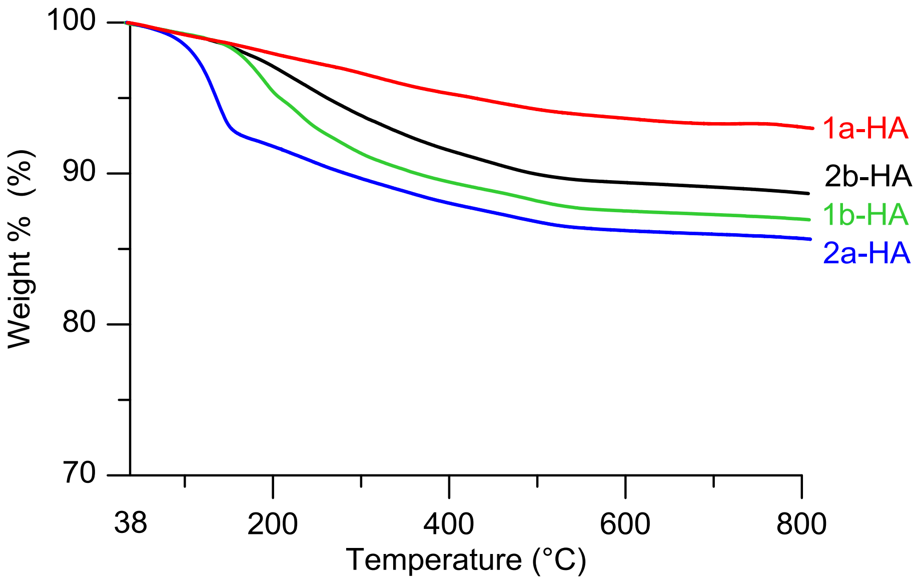

Figure SI-3. TGA scans of some samples containing different amounts of azetidinone as an example. **1a-HA** (red, 6.7 wt %); **2a-HA** (blue, 14.0 wt %); **1b-HA** (green, 12.6 wt %); **2b-HA** (black, 10.8 wt %). The shape of the weight loss curve is slightly different depending on the thermal stability of each organic molecule, but at over 550°C the weight is essentially stable.

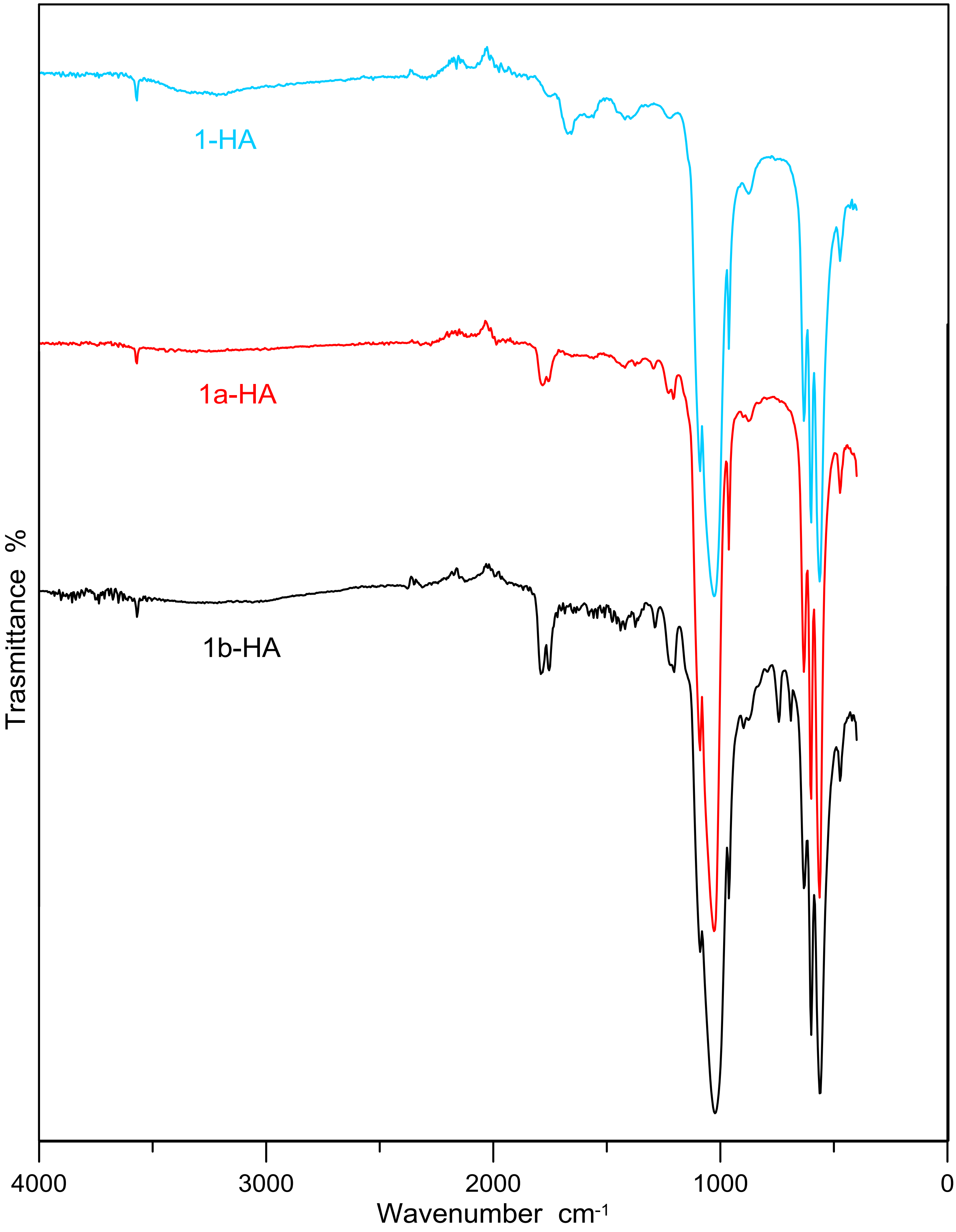

Figure SI-4A. ATR-FTIR spectra of samples **1-HA, 1a-HA**, **1b-HA**.

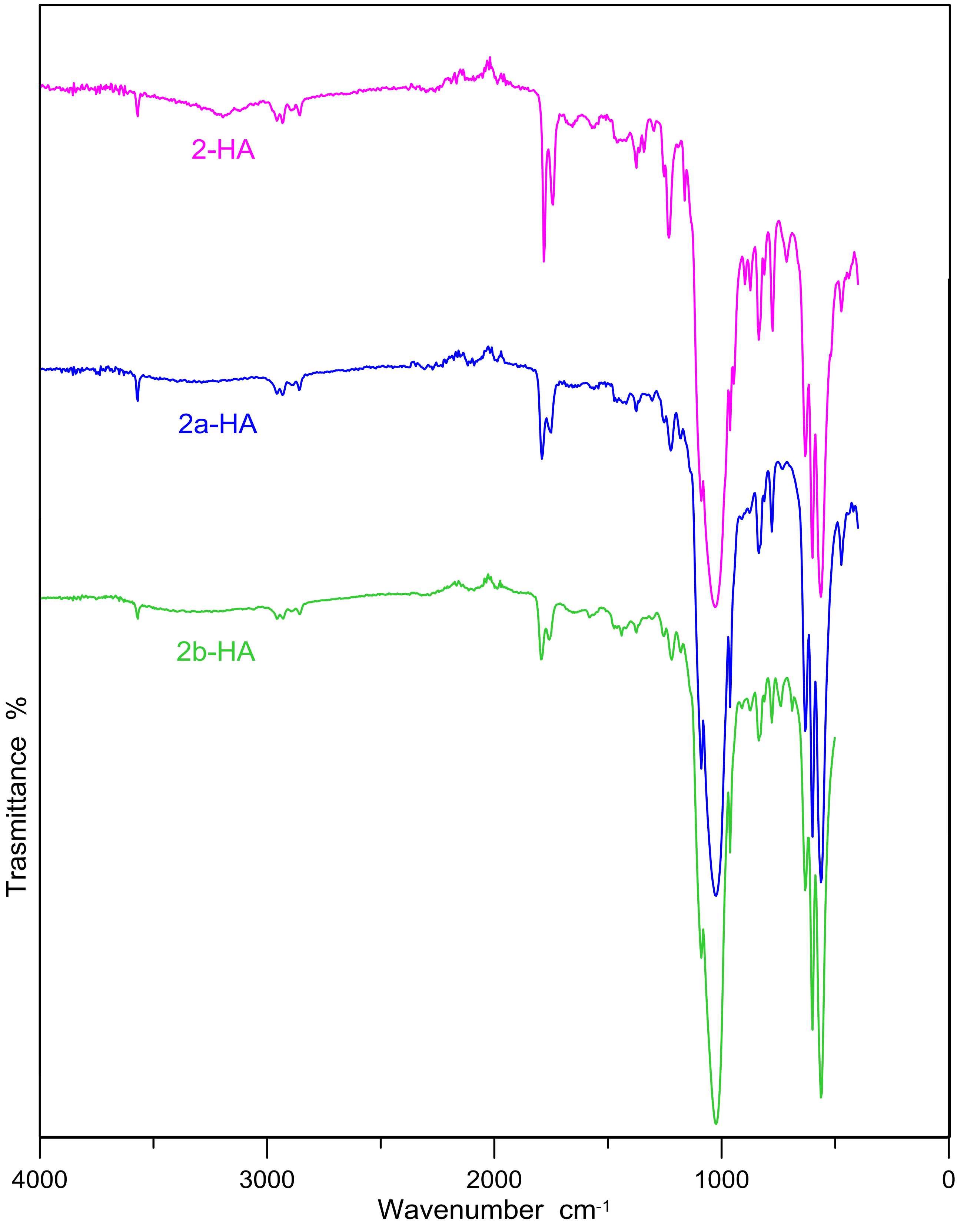

Figure SI-4B. ATR-FTIR spectra of samples **2-HA, 2a-HA**, **2b-HA**.

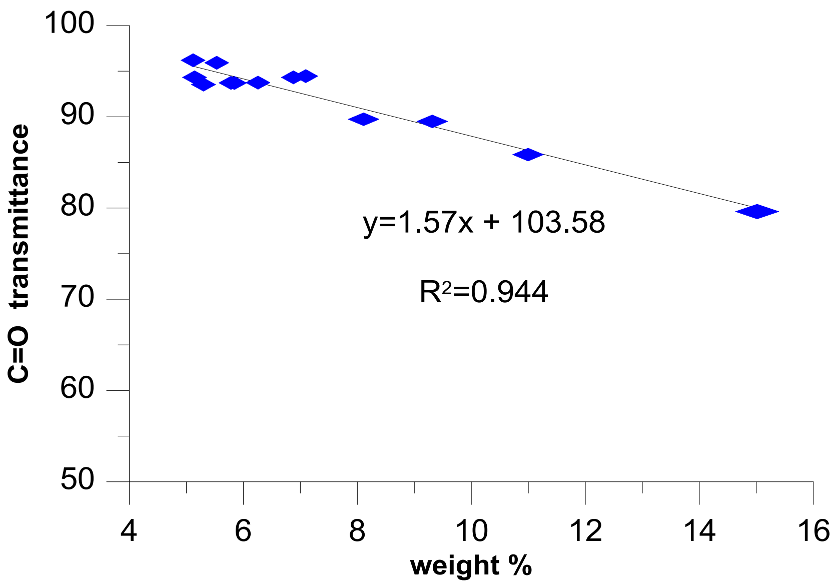

Figure SI-5. Correlation of azetidinone content evaluated by means of TGA analysis and ATR FT-IR transmittance of C=O signal for **2a-HA** sample. The intensity of the C=O stretching of the lactam at 1790 cm^{-1} was determined from transmittance values upon base line correction at 4000 cm^{-1} and normalization to the phosphate band at 1026 cm^{-1}. The diagonals of the rhombs are the standard deviation of the measurements.

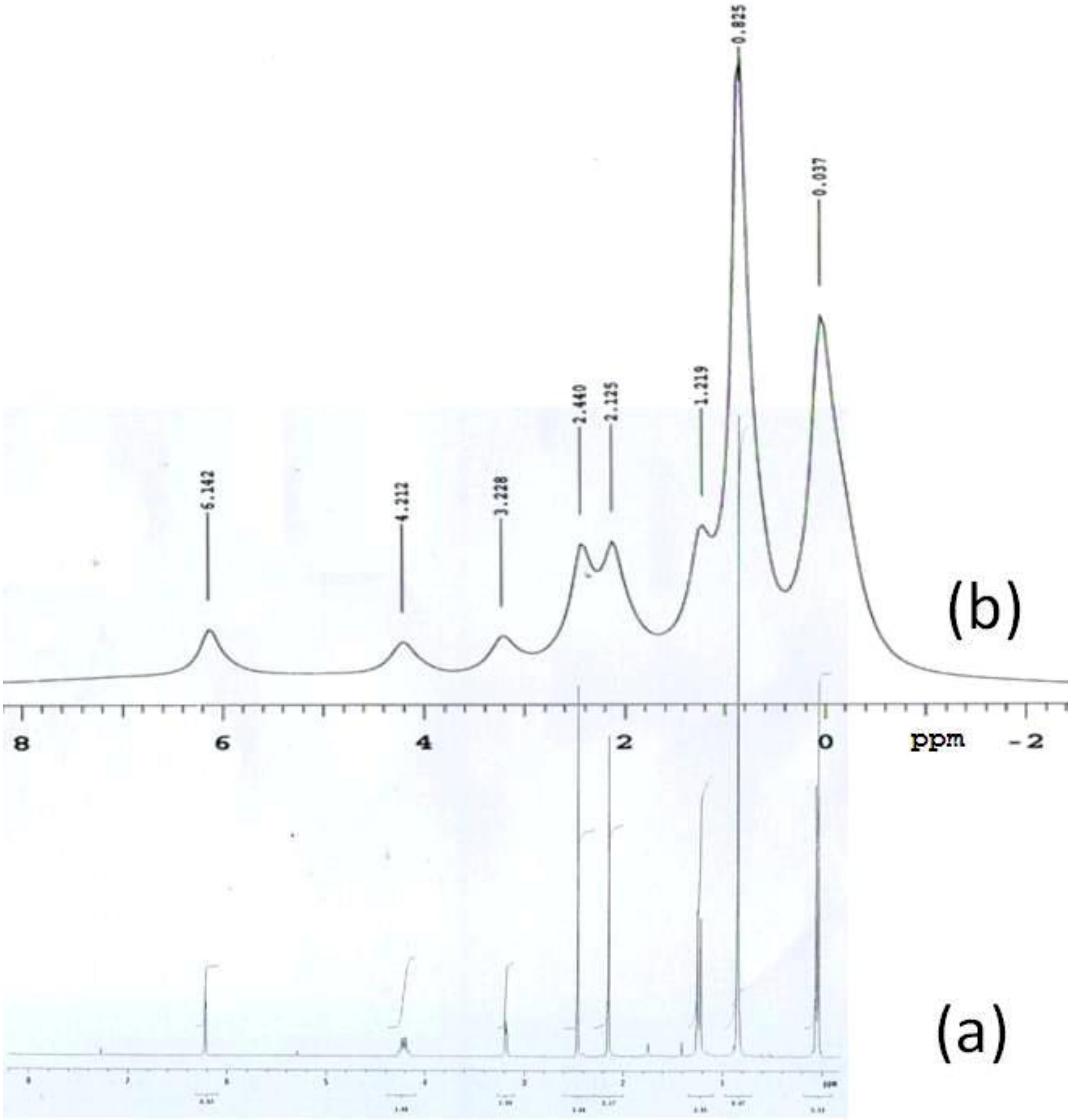

Figure SI-6. Comparison between the ^{1}H NMR spectra of **2a** in $CDCl_3$ solution (a), and **2a-HA** MAS-NMR in solid state of (b).

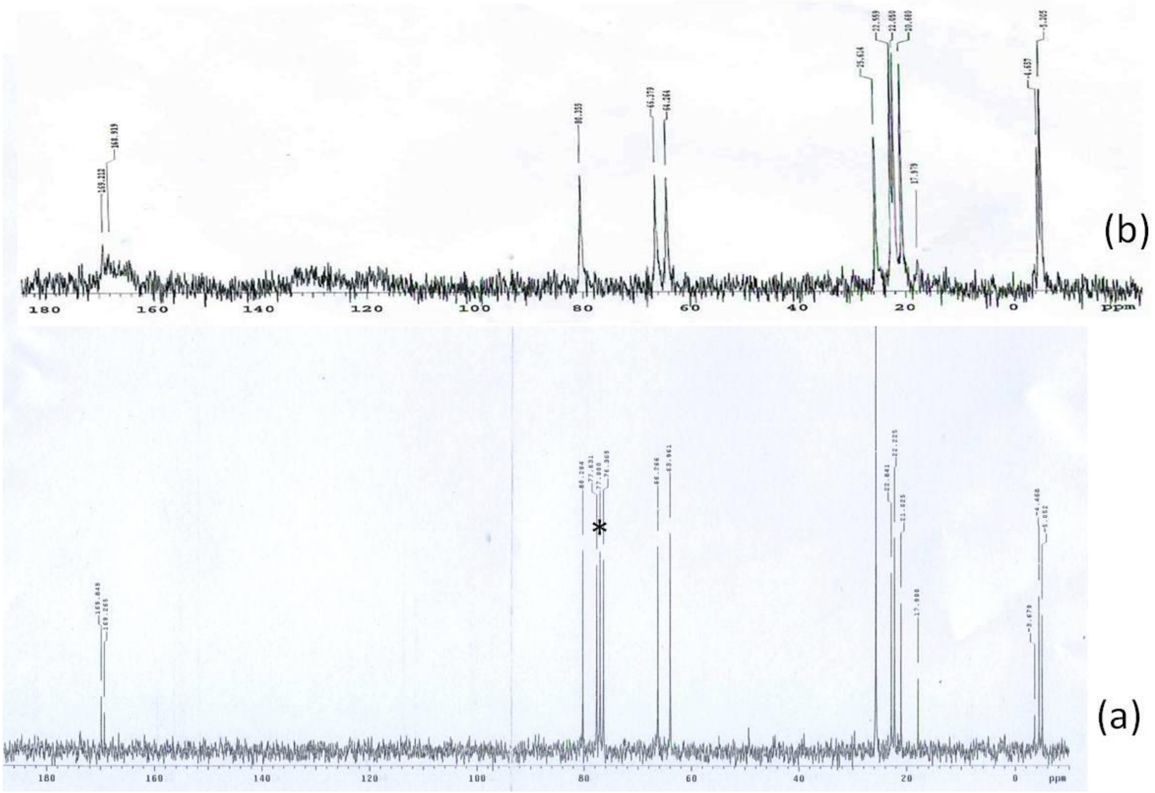

Figure SI-7. Comparison between the ^{13}C NMR spectra of **2a** in $CDCl_3$ solution (a), and **2a-HA** MAS-NMR in solid state (b). *Asterisk indicates $CDCl_3$ signals.

2. Materials and Methods

2.1 General Methods. All chemicals and solvents were of analytical grade; anhydrous solvents were obtained commercially and used without further drying. Azetidinones **1** and **2** are commercially available (Sigma-Aldrich) and used as such. Deionized water was obtained from a Millipore analytical deionization system (MilliQ). ATR-FTIR spectra were recorded on an Alpha FT IR Bruker spectrometer with platinum ATR single reflection diamond module. As reference, the background spectrum of air was collected before the acquisition of each sample spectrum. Spectra were recorded with a resolution of 4 cm^1, and 32 scans were averaged for each spectrum (scan range 4000–450 cm^1). TLC: Merck 60 F254 plates. Column chromatography: Merck silica gel 200-300 mesh. HPLC-MS: Agilent Technologies HP1100 instrument, equipped with a ZOBRAX-Eclipse XDB-C8 Agilent Technologies column, mobile phase: H_2O/CH_3CN, 0.4 mL/min, gradient from 30 to 80% of CH_3CN in 8min, 80% of CH_3CN until 25 min, coupled with an Agilent Technologies MSD1100 single-quadrupole mass spectrometer: full scan mode from m/z = 50 to 2600, scan time 0.1 s in positive ion mode, ESI spray voltage 4500 V, nitrogen gas 35psi, drying gas flow 11.5mL/min, fragmentor voltage 20 V. ^1H and ^{13}C spectra were recorded with an INOVA 400 or Gemini 300 instruments with a 5mm probe. All chemical shifts are quoted relative to deuterated solvent signals (δ in ppm and *J* in Hz).The solid state NMR spectra were obtained on a 500MHz Agilent DD_2 spectrometer by using a 22 μL rotor. The ^{13}C and ^1H spectra were recorded at a MAS frequency of 6kHz. TGA analysis was carried out heating under air stream from 40.0°C at 10.0°C/min in a TGA7 Perkin Elmer instrument. XRD patterns were collected by using a PANalytical X'PertPro diffractometer equipped with a fast solid state X'Celerator detector and a copper target (λ = 0.15418 nm). Data were acquired in the 9 – 60° 2θ interval, by collecting data for 50 s at each 0.05° step. For TEM investigations, a small amount of powder was dispersed in ethanol and submitted to ultrasonication. A drop of the calcium phosphate suspension was transferred onto holey carbon foils supported on conventional copper microgrids. A Philips CM 100 transmission electron microscope operating at 80 kV was used.

2.2 Synthesis of hydroxyapatite. It was carried out using CO_2-free distilled water in N_2 atmosphere [Bigi et al., 2004]. 50 ml of 1.08 M $Ca(NO_3)_2$ $4H_2O$ solution at pH adjusted to 10 with NH_4OH was heated at 90°C and 50 ml of 0.65 M $(NH_4)_2HPO_4$ solution, at pH 10 adjusted with NH_4OH, was added dropwise under stirring. The precipitate was maintained in contact with the reaction solution for 5 hours at 90°C under stirring, then centrifuged at 10,000 rpm for 10 minutes and repeatedly washed with CO_2-free distilled water. The product was dried at 37°C overnight.

2.3 Synthesis of azetidinones.

4-Acetoxy-1-(methylthio)-azetidin-2-one (**1a**): In a 50 mL 2-neck flask under nitrogen, Me_2S_2 (113 µL, 1.25 mmol) was added to anhydrous dichloromethane (DCM, 1 mL). The mixture was stirred at 0°C and a solution of SO_2Cl_2 (122 µL, 1.5 mmol) in anhydrous DCM (1 mL) was then added. After 15 min 4-acetoxy-azetin-2-one (129 mg, 1 mmol) was introduced followed by the addition of trimethylamine (TEA, 279 µL, 2 mmol). The consumption of the starting material was monitored by TLC analysis (AcOEt/Cyclohexane 3:7). The mixture was quenched with a saturated solution of NH_4Cl (2 mL) and extracted with EtOAc (3x10 mL). The organic layer were collected, dried over anhydrous Na_2SO_4, filtered and concentrated in vacuo. The crude was purified by flash-

chromatography (cyclohexane/EtOAc, 8:2) to finally afford the pure product as a yellow oil in a 77% yield. ^{1}H NMR (400 MHz, $CDCl_3$): δ = 2.15 (s, 3H), 2.47 (s, 3H), 3.03 (dd, *J* = 15.3, 1.6 Hz, 1H), 3.38 (dd, *J* = 15.3, 4.2 Hz, 1H), 6.10 (dd, *J* = 4.2, 1.6 Hz, 1H) ppm. ^{13}C NMR (100 MHz, $CDCl_3$): δ = 20.9, 22.8, 46.3, 78.8, 167.9, 170.1 ppm. IR (film): ṽ = 3341, 3200, 1790, 1752, 1393, 1213,755 cm^{-1} HPLC-MS: Rt = 2.25 min, m/z = 176 $[M+H]^+$, 198 $[M+Na]^+$.

4-acetoxy-1-(phenylthio)-azetidin-2-one (**1b**): In a 25 mL 2-neck flask under nitrogen, Ph_2S_2 (218 mg, 1mmol) in anhydrous DCM (1 mL) was introduced. The mixture was stirred at 0°C and a solution of SO_2Cl_2 (122 µL, 1.5 mmol) in anhydrous DCM (1 mL) was then added. After 15 min 4-acetoxy-azetidin-2-one (129 mg, 1 mmol) was introduced followed by the addition of TEA (279 µL, 2 mmol). The mixture was stirred at reflux for 2h. When TLC analysis (AcOEt/Cyclohexane, 2:8) indicated complete consumption of the starting material, the mixture was quenched with a saturated aqueous solution of NH_4Cl (2 mL) and extracted with EtOAc (3x10 mL). The collected organic solution was dried over Na_2SO_4, filtered and concentrated in vacuum. The crude was purified by flash-chromatography (cyclohexane/EtOAc, 8:2) to finally afford the product in a 87% yield (yellow oil). ^{1}H NMR (400 MHz, $CDCl_3$): δ = 1.99 (s, 3H), 3.10 (dd, *J* = 15.3, and 1.2 Hz, 1H), 3.42 (dd, *J* = 15.3, and 4.3 Hz, 1H), 6.19 (dd, *J* = 4.2, and 1.2 Hz, 1H), 7.50 – 7.27 (m, 5H) ppm. ^{13}C NMR (100 MHz, CDCl3): δ = 20.6, 46.2, 78.6, 128.6, 129.2, 136.0, 167.2, 170.0 ppm. IR (film): ṽ = 3059, 2923, 2850, 1785, 1754, 1585, 1355, 1287, 1154, 1047, 1000, 898, 740, 690 cm^{-1} HPLC-MS: Rt = 10.15min, m/z = 238 $[M+H]^+$, 260 $[M+Na]^+$

(2R, 3R)-3-(-1-(t-butyldimethysilyloxy)ethyl)-4-acetoxy-1-(methylthio)-azetidin-2-one (**2a**): In a 50 mL 2-neck flask under nitrogen, Me_2S_2 (90 µL, 1 mmol) was introduced in anhydrous DCM (1 mL). The mixture was stirred at 0°C and a solution of SO_2Cl_2 (41 µL, 0.5 mmol) in anhydrous DCM (1 mL) was then added. After 15 min **2** (287 mg, 1 mmol) was introduced followed by TEA addition (307 µL, 2.2 mmol). The mixture was stirred at reflux for 2 h. The consumption of the starting material was monitored by TLC analysis (AcOEt/Cyclohexane 3:7). The mixture was quenched with a saturated solution of NH_4Cl (2 mL) and extracted with EtOAc (3 x 10 mL). The organic layer were collected, dried over anhydrous Na_2SO_4, filtered and concentrated in vacuo. The crude was purified by flash-chromatography (cyclohexane/EtOAc, 9:1) to finally afford the pure product **2a** as yellow oil in a 84% yield. ^{1}H NMR (400 MHz, $CDCl_3$): δ = 0.03 (s, 3H), 0.05 (s, 3H), 0.84 (s, 9H), 1.26 (d, *J* = 6.4 Hz, 3H), 2.13 (s, 3H), 2.45 (s, 3H), 3.17 (dd, *J* = 2.9, 1.4 Hz, 1H), 4.21 (dq, *J* = 6.3, and 2.9 Hz, 1H), 6.21 (d, *J* = 1.3 Hz, 1H) ppm ^{13}C NMR (100 MHz, $CDCl_3$): δ = -5.1,-4.5, 17.9, 21.0, 22.2, 22.8, 25.7, 63.9, 66.3, 80.2, 169.3, 169.8 ppm. IR (film): ṽ = 2955, 2929, 2856, 1792, 1751, 1251, 837cm^{-1} HPLC-MS: Rt = 12.28 min, m/z = 356 $[M+Na]^+$, 234 $[M+H]^+$.

(2R,3R)-3-(-1-(t-butyldimethysilyloxy)ethyl)-4-acetoxy-1-(phenylthio)-azetidin-2-one (**2b**): In a 25 mL 2-neck flask under nitrogen Ph_2S_2 (218 mg, 1 mmol) in anhydrous DCM (1 mL) was introduced. The mixture was stirred at 0°C and a solution of SO_2Cl_2 (122 µL, 1.5 mmol) in anhydrous DCM (1 mL) was then added. After 15 min compound **2** (287 mg, 1 mmol) was introduced followed by the addition of TEA (279 µL, 2 mmol). The mixture was stirred at reflux for 2 h. When TLC analysis (AcOEt/Cyclohexane, 2:8) indicated a complete consumption of the starting material, the mixture was quenched with a saturated solution of NH_4Cl (2 mL) and extracted with EtOAc (3 x 10 mL). The organic extracts were collected, dried over Na_2SO_4, filtered and concentrated in vacuum. The

crude was purified by flash-chromatography (cyclohexane/EtOAc, 9:1) to finally afford the product (dark yellow oil) in a 75% yield. 1H NMR (400 MHz, $CDCl_3$): δ = -0.01 (s, 3H), 0.03 (s, 3H), 0.80 (s, 9H), 1.24 (d, *J* = 6.4 Hz, 1H), 1.94 (s, 3H), 3.26 (dd, *J* = 3.3, and 1.4 Hz, 1H), 4.21 (dq, *J* = 6.3, 3.4 Hz, 1H), 6.32 (d, *J* = 1.2 Hz, 1H), 7.24-7.49(m, 5H) ppm. ^{13}C NMR (100 MHz, $CDCl_3$): δ = -5.13, -4.52, 17.8, 20.6, 22.2, 25.6, 64.1, 66.1, 80.8, 128.2, 128.9, 129.1, 136.3, 169.1, 169.8 ppm.

Dansyl derivative of azetidinone **2**

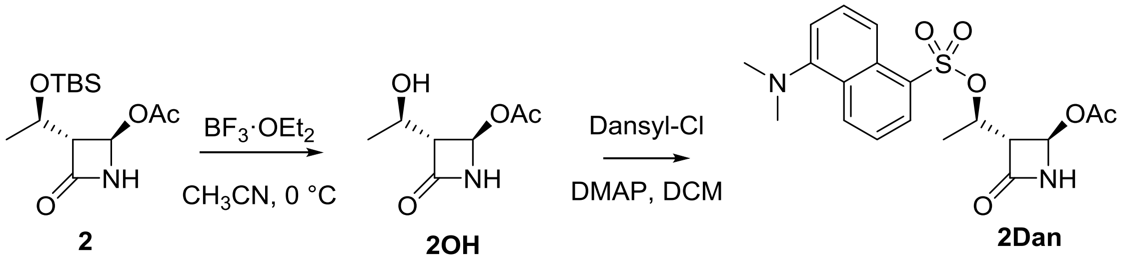

2Dan was prepared in a two steps synthesis starting from beta-lactam **2.** Compound **2OH** was prepared according to the procedure reported in Galletti et al. ChemMedChem 2011, 6, 1919-1927 with $BF_3 \cdot OEt_2$.

(2R,3R)-3-((R)-(((5-(dimethylamino)naphthalenyl)sulfonyl)oxy)ethyl)-4-oxoazetidin-2-yl acetate (**2Dan**): in a 10mL 2-necks flask, to a solution of **2OH** (87mg, 0.5mmol) in 5mL of anhydrous DCM were added dansyl chloride (148mg, 0.55mmol) and DMAP (32mg, 0.75mmol). The reaction was followed by TLC (cyclohexane/EtOAc 50/50) and after 24hours stirring was directly concentrated in vacuum. The crude product was purified by flash chromatography (cyclohexane/EtOAc 70/30) to afford TM in 36% yield (73mg) as a yellow-green oil. Rf 0.32 (cyclohexane/EtOAc 50/50), $[\alpha]_D$ 25 = -17.83° (c = 13.27, DCM). ^1H NMR (400 MHz, $CDCl_3$): δ 1.38 (d, *J* = 6.0Hz, 3H, $MeCHOSO_2$), 2.00 (s, 3H, Ac), 2.93 (s, 6H, NMe_2), 3.32 (d, *J* = 8.8Hz, 1H, C*H*CHOAc), 4.81 (dquartet, *J* = 6.4, 8.4Hz, 1H, $CHOSO_2$), 5.25 (s, 1H, CHC*H*OAc), 6.34 (s, 1H, NH), 7.23 (d, *J* = 8.0Hz, 1H, Ar), 7.56-7.62 (m, 2H, Ar), 8.26 (d, *J* = 8.8Hz, 1H, Ar), 8.30 (d, *J* = 7.6Hz, 1H, Ar), 8.67 (d, *J* = 8.0Hz, 1H, Ar) ppm. ^{13}C NMR (50.3 MHz, $CDCl_3$): δ 19.4, 20.5, 45.3 (2C), 62.0, 75.6, 75.7, 115.5, 119.3, 123.0, 128.5, 129.6, 129.7, 130.2, 131.7, 131.8, 151.7, 163.3, 170.5 ppm. IR: ṽ = 3331, 2937, 1785, 1753, 1359, 1232, 1177, 1140 cm^{-1} HPLC-MS: Rt = 8.74min; m/z = 407 (100) $[M+H]^+$, 813 $[2M+H]^+$, 435 (25) $[2M+Na]^+$

2.4 Loading of Azetidinones The loading of azetidinones on HA was conducted in H_2O (method A) or H_2O/organic solvent mixtures (method B), see below. Loading processes were set up in a parallel-synthesis fashion with a Carousel 6 reaction station using two necks round bottom flasks (50 mL) with a water-cooled aluminum head. This apparatus is well suited to keep constant some experimental conditions, such as stirring and warming, important parameters in heterogeneous phase processes, and, moreover, it improved and speeded up optimization steps. For loading on HA in water alone was used method A, in the presence of a co-solvent method B, instead. *Method A:* 200 mg of HA nanoparticles were suspended in 2 mL of H_2O and warmed at 40°C under magnetic stirring. Azetidinone (50 mg) was added in one portion to the suspension which was then warmed up to 70°C. Reaction mixtures were controlled via TLC on the supernatant solution to

monitoring the starting azetidinone disappearance. After 4 h the mixture was quantitatively transferred with 1 mL of H_2O/MeCN (1:1) in an open test tube and centrifugated for 1 min at 700 rpm. The solid phase was perfectly separated and the supernatant aqueous phase was collected and extracted with dichloromethane (1x3 mL). The aqueous and dichloromethane phases were separately evaporated and analyzed to quantify the unloaded azetidinone and its distribution in the two phases. Data were expressed as loading efficiency % back-calculated from the added up residues obtained in DCM and H_2O in comparison with the amount of azetidinones in the loading solution by the equation:

$$LE = \frac{[\mathrm{A} - (\mathrm{rw} + \mathrm{rDCM})]}{\mathrm{A}} * 100$$

where:
LE = loading efficiency %; A = amount (g) of azetidinone in the loading solution; rw = residue (g) of azetidinone in water extract; rDCM = residue (g) of azetidinone in dichloromethane(DCM) extract.

The solid functionalized HA material was oven dried at 35°C for 24 h, and kept in dessicator ($CaCl_2$) for 24 h before the analyses. *Method B:* 200 mg of HA nanoparticles were suspended in 1 mL of H_2O and warmed at 40°C under magnetic stirring, then azetidinone (50 mg) was solubilized in 1 mL of organic solvent and added to the suspended HA, then the mixture was warmed at 70°C under stirring for 4 h. Reaction mixtures were controlled via TLC on the supernatant solution to monitoring the starting azetidinone disappearance. The work-up procedure was as for Method A.

Loading amount of the azetidinone molecules on HA was evaluated through thermogravimetric analysis as difference between the total weight loss measured between 38 and 800°C for each loaded sample and that measured for pristine HA. Moreover, the determination was also performed through the evaluation of the intensity of the adsorption band of C=O at 1790 cm^{-1}

2.5 In vitro release. The release profiles of azetidinones loaded on HA were investigated in H_2O Milli Q, buffer phosphate (0.1 M, pH = 7.4), and buffer acetate (0.1 M, pH=5). Samples of azetidinones **1a-HA** (8.1% of loaded azetidinone, TGA measurement), **1b-HA** (12.6 %), **2a-HA** (15.7 %), and **2b-HA** (10.85 %) were used for the in vitro release study. In a 10 mL test tube an azetidinone-HA sample (50 mg) was suspended in 2.5 mL of the aqueous solutions H_2O Milli Q, or buffer phosphate (0.1 M, pH = 7.4), or buffer acetate (0.1 M, pH=5). Experiments were conducted at 37°C in thermostat with sampling and refresh of the aqueous solution after 1, 2, 3, 6, 8, 24, 30 h. At each time point, the solution was centrifugated (1 min. 700 rpm) and the supernatant was separated and the released concentration of an azetidinone was determined by HPLC-UV analysis. The solid was incubated again with a fresh solution of the specific medium (2.5 mL).The release of samples **1a-HA** and **2a-HA** were also studied in buffer acetate at pH = 5 by a once-a-day refresh for 9 days with the procedure and analysis as above described. Linear calibration curves for the HPLC-UV analysis of azetidinones in surnatant solutions were established at 210 nm (column: Phenomenex Gemini C18, 3 µm, 100 x 3mm, flow 0.4 ml/min, T = 30°C); parameters for **1a** were R_t = 3.9 min H_2O/CH_3CN = 80:20 in the region of concentration from 3 to 0.15 mM; for **1b** were R_t = 5.3 min H_2O/CH_3CN = 60:40 in the region of concentration from 2.5 to 0.05 mM; for **2a** were R_t =

12.2 min from H_2O/CH_3CN = 80:20 to 20:80 in 8 min, in the region of concentration from 1 to 0.225 mM; for **2b** were R_t = 7.1 min H_2O/CH_3CN = 30:70 in the region of concentration from 1 to 0.05 mM.

2.6 Cytotoxicity.

2.6.1 In vitro tests. MG63 osteoblast-like cell line was expanded in Dulbecco' Modified Eagle Medium (DMEM, Sigma, UK) supplemented with 10% FCS, and antibiotics (100 U/ml penicillin, 100 μg/ml streptomycin). Cells were detached from culture flasks by trypsinization, and cell number and viability were checked by trypan blue dye exclusion test. Cells were plated at a density of $2x10^4$ cells/ml in 24-well plates onto sterile samples of unloaded hydroxyapatite (HA), HA loaded with the monocyclic azetidinones **1a-HA**, **1b-HA**, **2a-HA**, and **2b-HA,** and in wells for negative (CTR–, DMEM only) and positive (CTR+, DMEM + 0.05% phenol solution) controls for cytotoxicity tests (according to UNI EN ISO 10993-5, Biological evaluation of medical devices – Part 5. Tests for in vitro cytotoxicity). Plates were cultured in standard conditions, at 37 ± 0.5°C with 95% humidity and 5% ± 0.2 CO_2 for 72 h. Cytotoxicity tests were performed after 48 and 72 h of culture. Cell proliferation and viability was assessed by WST1 (WST1, Roche Diagnostics GmbH, Manheim, Germany) colorimetric reagent test. The assay is based on the reduction of tetrazolium salt to a soluble formazan salt by a reductase of the mitochondrial respiratory chain, active only in viable cells. 100 μl of WST1 solution and 900 μl of medium (final dilution: 1:10) were added to the cell monolayer, and the multi-well plates were incubated at 37°C for a further 4 h. Supernatants were quantified spectrophotometrically at 450 nm with a reference wavelength of 625 nm. Results of WST1 are reported as optical density (OD) and directly correlate with the cell number. At the end of experimental time the supernatant was collected from all wells to detect Lactate Dehydrogenase (LDH, enzyme-kinetic test, Roche Diagnostics GmbH) release: 100μl of reagent were added to 100μl of cell supernatant in a 96-wells plate; after 30min of incubation at room T in the dark, samples were evaluated by spectrophotometer at 490/655nm.

2.6.2 Statistical analysis. The statistical evaluation of data was performed using the software package SPSS/PC+StatisticsTM 23.0 (SPSS Inc., Chicago, IL USA). The study is the results of three independent experiments and data are reported as mean ± standard deviations (SD) at a significance level of $p<0.05$. After having verified normal distribution and homogeneity of variance, a one-way ANOVA was done for comparison between groups. Finally, post hoc multiple comparison test and Pearson correlation test were performed to detect significant differences among groups and controls.

2.7 Antibacterial susceptibility testing

2.7.1 Bacterial strains. The *in vitro* effect of the HA nanocrystals loaded with the monocyclic azetidinones was evaluated against Gram-positive and Gram-negative reference bacterial strains: *Staphylococcus aureus* (ATCC 25923), *Escherichia coli* (ATCC 25922). In addition, clinical isolates obtained from surgical bone biopsies were included in the study and they were categorized based on their antimicrobial susceptibility to methicillin. The tested strains were isolated on BD Columbia Agar with 5% sheep blood (Becton Dickinson, Germany) and confirmed by MALDI-TOF MS (Bruker Daltonik, Germany) (Croxatto et al. 2012). Their susceptibility was analyzed by the Vitek2 semi-automated system (bioMerieux, France) and interpreted following EUCAST guidelines. MRSA strains were confirmed by growth on BD oxacillin screen agar (Becton Dickinson, Germany), as in

the clinical microbiology laboratory resistance to oxacillin is the marker for detecting methicillin resistance (Brown et al., 2005). SCV phenotypic characterization was carried out by identification of very small pinpoint colonies on blood agar plate following 48h of growth.

2.7.2 Kirby-Bauer (KB) disk diffusion method. The assay was performed following the requirements of the CLSI 2006) and allowed to measure the diameter of the inhibition zone (in millimeter) surrounding the azetidinone-HA samples. Briefly, the surface of MH agar (MHA) (Sigma-Aldrich) was inoculated with a bacterial suspension at 0.5 McFarland, prepared in sterile 0.9% saline solution. Gamma rays sterilized disk samples were placed on the agar plates and incubated at 37°C for 24 hours when the reading and interpretation of zones of inhibition were carried out. Plates were further incubated up to 7 days to check the inhibitory effects over the time.

2.8 References

Bigi, A., Boanini, E., Gazzano, M., Kojdecki, M. A., Rubini, K. Microstructural investigation of hydroxyapatite–polyelectrolyte composites. *J. Mater. Chem.* **14,** 274-279 (2004).

Brown, D.F.J. *et al.* Guidelines for the laboratory diagnosis and susceptibility testing of methicillin-resistant Staphylococcus aureus (MRSA) *J. Antimicrobial Chemot.* **56**, 1000-1018 (2005).

Croxatto, A., Prod'hom, G., Greub, G. Applications of MALDI-TOF mass spectrometry in clinical diagnostic microbiology *FEMS Microbiol. Rev.* **36,** 380-407 (2012).

Galletti, P. *et al.* Antibacterial agents and cystic fibrosis: synthesis and antimicrobial evaluation of a series of N-thiomethylazetidinones. *ChemMedChem* **6**, 1919-1927 (2011).
